# Phenotypic dichotomy in *Crotalus durissus ruruima* venom and potential consequences for clinical management of snakebite envenomations

**DOI:** 10.1371/journal.pntd.0013296

**Published:** 2025-08-01

**Authors:** Mônica Colombini, Anderson M. Rocha, Luciana A. Freitas-de-Sousa, Alison F. A. Chaves, Solange M. T. Serrano, Vinicius C. Souza, Vincent L. Viala, Inácio L. M. Junqueira-Azevedo, Felipe A. Cerni, Marco A. Sartim, Jacqueline A. G. Sachett, Wuelton M. Monteiro, Felipe G. Grazziotin, Fan Hui Wen, Manuela B. Pucca, Ana M. Moura-da-Silva

**Affiliations:** 1 Laboratório de Imunopatologia, Instituto Butantan, São Paulo, Brazil; 2 Departamento de Ensino e Pesquisa, Fundação de Medicina Tropical Doutor Heitor Vieira Dourado, Manaus, Brazil; 3 Escola Superior de Ciências da Saúde, Universidade do Estado do Amazonas, Manaus, Brazil; 4 Laboratório de Toxinologia Aplicada, Centro de Toxinas, Resposta Imunológica e Sinalização Celular (CeTICS), Instituto Butantan, São Paulo, Brazil; 5 Laboratório de Bioquímica, Instituto Butantan, São Paulo, Brazil; 6 Instituto de Ciências Biológicas, Universidade Federal de Roraima, Boa Vista, Brazil; 7 Faculdade de Ciências Farmacêuticas, Universidade Federal do Amazonas, Manaus, Brazil; 8 Laboratório de Coleções Zoológicas, Instituto Butantan, São Paulo, Brazil; 9 Processamento de Plasmas Hiperimunes Indiretos, Fundação Butantan, São Paulo, Brazil; 10 Faculdade de Ciências Farmacêuticas, Unesp, Araraquara, Brazil; Lancaster University Faculty of Health and Medicine, UNITED KINGDOM OF GREAT BRITAIN AND NORTHERN IRELAND

## Abstract

**Background:**

Phenotypic polymorphism in rattlesnake venoms is well-documented, with a dichotomy between hemorrhagic (Type I) and neurotoxic (Type II) venoms. In South America, the Type II phenotype is predominant; however, evidence of Type I venom in *Crotalus durissus ruruima* raises concerns about the efficacy of the *Crotalus* antivenom, which is prepared only with Type II venoms. Consequently, the *Bothrops*-*Crotalus* antivenom has been proposed as an alternative treatment for envenomation by Type I venoms.

**Methodology/Principal findings:**

This study characterizes the dichotomy of *C. d. ruruima* venom by analyzing the structure of isoforms differentially expressed in Type I and Type II venoms, assessing their biological activities, and evaluating the implications for snakebite clinical management in Roraima State (northern Brazil). Four toxins were differentially expressed between Type I and Type II venoms: two PIII-class SVMPs, predominantly found in Type I venoms, associated with proteolytic and hemorrhagic activity; and two PLA_2_s, corresponding to Crotoxin A and B chains, prevalent in Type II venoms and related to elevated phospholipase A_2_ activity, myotoxicity, and increased lethality. The structure of Crotoxin chains was well conserved compared to *C. d. terrificus* Crotoxin. However, the SVMP sequences exhibited multiple substitutions in functional and immunoreactive regions compared to Bothropasin, resulting in low hemorrhagic activity and limited reactivity/neutralization by the *Bothrops* antivenom. Conversely, the *Crotalus* antivenom reacted with high antibody titer and neutralized all activities of both venom subtypes, except for the low hemorrhagic activity induced by Type I venoms.

**Conclusions/Significance:**

The efficacy of *Bothrops* antivenom in snakebites caused by rattlesnakes with Type I venoms remains uncertain. We advocate for a clinical study in Roraima to assess patient outcomes and benefits of *Bothrops*-*Crotalus* versus *Crotalus* antivenoms for these accidents. Meanwhile, administering *Bothrops*-*Crotalus* antivenom may be acceptable; however, caution is needed regarding the use of heterologous *Bothrops* antibodies, which have limited efficacy in treating *Crotalus* envenomation.

## Author summary

Rattlesnake venoms exist in two forms: hemorrhagic (Type I) and neurotoxic (Type II). In South America, neurotoxic venoms are predominant; however, evidence of hemorrhagic venoms in rattlesnakes from northern Brazil, Guyana, and Venezuela raises concerns about treating snakebite patients in the region. This study details the presence of both hemorrhagic and neurotoxic venoms in *Crotalus durissus ruruima*, the rattlesnake found in Roraima State, Brazil. Type I and Type II venoms differ in their toxin composition, which explains the specific toxicity of each type. In experimental models, antivenoms indicated for treating rattlesnake envenomation (*Crotalus* antivenom) showed a high antibody titer and neutralized toxic activities of both venom subtypes; however, it did not reduce the hemorrhagic activity induced by Type I venoms, which was diminished by *Bothrops* antivenom. Our data support previous indications that *Bothrops-Crotalus* antivenoms could be the preferred treatment for patients in northern Brazil. However, clinical studies reporting the benefits of the combined antivenoms remain underreported, and clinical studies in Roraima to assess patient outcomes and the effectiveness of *Bothrops-Crotalus* versus *Crotalus* antivenoms for this envenomation are strongly recommended.

## Introduction

Snake envenomation is a complex medical emergency that primarily affects low-income populations in rural areas of tropical countries. According to the Brazilian Ministry of Health [1], the incidence rate of snakebites in Brazil in 2022 was 14.6/100,000 inhabitants, with 34.8% of cases occurring in the country’s northern region, where the Amazon rainforest predominates. This region had an incidence rate of 55.3/100,000 inhabitants, approximately 4.1 times the national average. Roraima has the highest snakebite incidence rate among northern Brazilian states (68.6/100,000 inhabitants) and the highest lethality rate in the country (1.1% compared to the national average of 0.3%). This state is home to diverse indigenous ethnic groups, who comprise 11% of the population, in which the incidence of snakebites is even higher, reaching up to 12 times that of the general population [2]. The South American pit viper *Bothrops atrox* is the leading cause of snakebites in the Amazon region [3]. However, in Roraima, a rattlesnake subspecies, *Crotalus durissus ruruima,* accounts for 10.9% of cases [4]. *C. d. ruruima* inhabits the savannahs of Roraima State, southern Venezuela, and parts of Guyana at the northern extreme of South America [4].

The genus *Crotalus* (Viperidae) comprises approximately 55 species, some further divided into subspecies (http://www.reptile-database.org). *Crotalus durissus* includes 11 subspecies distributed across South America, with six subspecies occurring in Brazil (*C. d. durissus, C. d. cascavella, C. d. collilineatus, C. d. marajoensis, C. d. ruruima,* and *C. d. terrificus*) [5]. The venom of viperids is rich in proteolytic enzymes, particularly Snake Venom Metalloproteinases (SVMPs). This also applies to rattlesnakes; however, some species have undergone evolutionary shifts in venom composition, resulting in changes in pharmacological effects. Notably, the Mojave (*C. scutulatus*) and South American (*C. durissus*) rattlesnakes evolved potent neurotoxic venoms, in which Crotoxin-like toxins are dominant, lacking the hemorrhagic effects typical of rattlesnake bites [6,7]. Consequently, *Crotalus* venoms are classified into two types: Type I venoms, which are hemorrhagic with high expression of PIII-class SVMPs, leading to substantial tissue damage but low systemic toxicity; and Type II venoms that are highly neurotoxic, with reduced SVMP expression and increased presence of Crotoxin-like toxins [8].

The distribution of rattlesnake venom phenotypes across the Americas is intriguing. In North America, most rattlesnakes have Type I venoms, while a few species, such as *C. scutulatus, C. tigris*, and *C. horridus,* exhibit Type II venoms [9–12]. The venom dichotomy in the Mojave rattlesnake (*C. scutulatus*) has been extensively studied, revealing that both phenotypes occur in adult snakes, including individuals with intermediate phenotypes in regions of inferred introgression, in which both SVMPs and Crotoxins are expressed [13]. Some species may exhibit both venom phenotypes during ontogeny, with juveniles expressing Type II venoms and switching to Type I as adults [14–16]. A similar pattern is observed in Central America and the northern part of South America, where adult *C. simus* from Costa Rica and Guatemala and *C. cumanensis* from Venezuela exhibit Type I venoms, [17–19]. In contrast, the venoms of most of the South American *C. durissus* subspecies present low or lack metalloproteinase-related proteolytic and hemorrhagic activities [20–22]. These venoms are classified as Type II, characterized by the presence of Crotoxin, the neurotoxic and myotoxic phospholipases A_2_ (PLA_2_) that can lead to death due to renal or respiratory failure [23]. However, some reports suggest that the venom of *C. d. ruruima* may be classified as Type I, similar to the Venezuelan and Central American species *C. cumanensis* and *C. simus* [18]. The presence of Type I venom phenotype in *C. d. ruruima* may be associated with its shared habitats and the geographical proximity to regions in Venezuela and Central America, where adult rattlesnakes predominantly exhibit Type I venoms.

Despite the challenges posed by snakebites in Roraima, studies on the venom composition and pharmacological properties of *C. d. ruruima* venom remains limited. Some reports suggest an association between venom color and functional activities [2,22,24,25], with yellow venoms exhibiting higher proteolytic and hemorrhagic activities than white venoms. However, in a study analyzing six individuals, three of each color pattern, only one of the three yellow venoms exhibited hemorrhagic activity [22]. This finding suggests that the expression of the Type I phenotype is highly variable among individuals and unrelated to venom color. In fact, it is widely accepted that the yellow coloration of some venoms results from the presence of L-amino acid oxidase (LAO) and the pigment riboflavin as a cofactor, which is not associated with proteolytic activity.

Differences in the composition of *C. d. ruruima* venom, compared to other South American *C. durissus* subspecies, may have significant clinical implications for snakebite treatment in Roraima, where Brazil’s highest lethality rates are recorded. In Brazil, antivenoms are produced by immunizing horses with venoms from *C. d. terrificus* and *C. d. collilineatus*, both of which exhibit Type II venom phenotypes, raising uncertainties about whether the *Crotalus* antivenom produced in Brazil effectively neutralizes the hemorrhagic effects of Type I venoms [22,24]. However, despite this evidence and the relevance of understanding venom composition, only one C. *d. ruruima* venom proteome has been published [17]. In this study, based on a pool of six captive specimens with white venoms, Crotoxin was the dominant venom component, accounting for more than 80% of identified peptides [17], and the Type I venom phenotype was not identified.

These findings support our hypothesis of individual variation in the Type I/Type II venom phenotypes of *C. d. ruruima* and highlight the potential consequences for antivenom efficacy in the Roraima region. Therefore, a thorough evaluation of *C. d. ruruima* venom, its toxicity, and reactivity with antivenoms are urgently needed. In this study, we address this gap by providing a comprehensive characterization of venom composition, toxic activities, and antivenom neutralization, considering venoms from specimens collected across different areas of Roraima, from distinct ontogenetic stages, and exhibiting white or yellow venoms.

## Methods

### Ethics statements

The protocols used in this study have been submitted and approved by the Animal Ethical Use Committee of the Instituto Butantan (CEUA) under certificate nº 4222230223, issued on June 21, 2023 for animal studies. Snake collections for venom extraction were carried out under ICMBio/SISBIO permits 79102–4 and SISGEN AB5B495.

### Snakes and venoms

Nineteen specimens of *Crotalus durissus ruruima* were collected under ICMBio/SISBIO permit 79102–4 and SISGEN registration AB5B495, primarily in the northeastern region of Roraima State, Brazil. For each specimen, sex, ontogenetic stage, snout-vent length (SVL), venom coloration, and the municipality of collection were recorded (Table 1). The ontogenetic stage was determined by the biologist and co-author, Anderson M. Rocha, based on his extensive field experience in northern Brazil. Classification also followed standard herpetological criteria, considering morphological parameters such as SVL, body mass, head proportions, and rattle segment count. Specimens were classified as juveniles or adults based on these features, especially body size (>550 mm for adults and ≤550 mm for juveniles), coloration, and rattle development following widely accepted protocols for rattlesnake ontogeny.

**Table 1:** Description of the snake specimens used in this study.

Snake number	Venom color	Sex	Stage	SVL (mm)	City
Cdr 01	White	Female	Adult	1050	Boa Vista
Cdr 02	Yellow	Male	Adult	1250	Bonfim
Cdr 03	White	nd	Juvenile	550	Cantá
Cdr 04	Yellow	Female	Adult	1000	Bonfim
Cdr 05	Yellow	Female	Adult	1200	Bonfim
Cdr 06	White	Male	Adult	1000	Cantá
Cdr 07	White	Female	Juvenile	540	Normandia
Cdr 08	Yellow	Male	Adult	670	Bonfim
Cdr 09	Yellow	Male	Adult	780	Bonfim
Cdr 10	White	nd	Juvenile	350	Bonfim
Cdr 11	Yellow	Male	Adult	600	Bonfim
Cdr 12	Yellow	Male	Juvenile	410	Bonfim
Cdr 13	Yellow	Male	Adult	660	Bonfim
Cdr 14	White	Male	Adult	610	Boa Vista
Cdr 15	Yellow	Female	Adult	810	Amajari
SB 0831	Yellow	Male	Adult	nd	Boa Vista
SB 0833	Yellow	Male	Juvenile	505	Bonfim
SB 0834	White	Male	Adult	570	Boa Vista
SB 1130	Yellow	nd	Adult	nd	Boa Vista

SVL - snout-vent length; nd - Not determined at the time of collection.

### Characterization of venom composition

Individual or pooled venom samples (2 mg) were first subjected to reverse-phase chromatography in HPLC (Shimadzu, Japan) by standard protocols as previously described [26]. Following that, proteomic analysis was carried out with pooled or individual venom samples (30 μg) according to the methods used in our laboratory [27]. Briefly, trypsin-digested proteins were submitted to LC − MS/MS analyses in a Vanquish Neo (Thermo Scientific) UPLC system coupled to an Orbitrap Exploris 480 mass spectrometer (Thermo Fisher Scientific) under the same conditions described previously [26]. MS/MS samples were analysed using MSFragger (The Nesvizhskii Lab, 4237 Medical Science I, Ann Arbor, MI 48109). MSFragger was set up to search a reverse concatenated in-house database consisting of the protein sequences predicted from a non-redundant master set containing venom toxins annotated from the transcriptome of venom glands from SB831, SB833, SB834, and SB1130 specimens, assuming the digestion by trypsin. MSFragger was searched with a fragment ion mass tolerance of 20 PPM and a parent ion tolerance of 20 PPM. The oxidation of methionine and carbamidomethyl of cysteine were specified in MSFragger as varied and fixed modifications, respectively. Scaffold (version Scaffold_5.3.3, Proteome Software Inc., Portland, OR) was used to validate MS/MS-based peptide and protein identifications. Peptide identifications were accepted if they could be established at greater than 95,0% probability by the Percolator posterior error probability calculation [28]. Protein identifications were accepted if they could be established at greater than 99,9% probability and contained at least two identified peptides. Protein probabilities were assigned by the Protein Prophet algorithm [29]. Proteins that contained similar peptides and could not be differentiated based on MS/MS analysis alone were grouped to satisfy the principles of parsimony. The Normalized Total Spectra Counting (NTSC) was used to estimate the abundance of independent proteins and toxin groups. Mass spectrometry data were deposited via the PRIDE [30] repository and are available via ProteomeXchange with the identifier PXD060738. For the construction of the non-redundant transcriptome master set, cDNA library construction, sequencing, assembly and annotation were carried out as previously described [26]. Raw transcriptomic data are available at NCBI’s GenBank under Bioproject accession number PRJNA1188107 and Biosample accession numbers SRR31885731, SRR31885732, SRR31885733, and SRR31885734.

### Functional tests

#### *In vitro* tests.

Snake venom metalloproteinases (SVMP), snake venom serine proteinases (SVSP), and phospholipase A_2_ (PLA_2_) enzymatic activities of venom samples were assayed using the synthetic substrates following the experimental protocols previously described [31]. Briefly, SVMP activity was assessed by Fluorescence Resonance Energy Transfer substrate Abz-AGLA-EDDnp (GenOne Biotechnologies), and the enzymatic reactions were monitored in a SpectraMax M2 fluorimeter (Molecular Devices) with excitation at 320 nm and emission at 420 nm, at 37 °C in kinetic mode over 10 min with a read range of 1 min. The results were expressed in RFU/min/µg. The SVSP activity was determined using the chromogenic synthetic substrate benzoyl-arginyl-p-nitroanilide (L-BAPNA) (Sigma-Aldrich) incubated with venom samples (5µg) at 37 °C for 40 min. Hydrolysis was measured spectrophotometrically at 405 nm, and activity was expressed as absorbance at 405 nm/min/mg of venom. The PLA_2_ activity of venom samples (5µg) was assayed using the synthetic substrate 4-nitro-3- [octanoyloxy] benzoic acid (Enzo Life Sciences) at a final concentration of 320 µM. The plates were incubated for 40 min at 37 °C, and hydrolysis values were determined by measuring the absorbance at 425 nm and expressed as absorbance per minute per milligram of venom. The results represent the mean ± SD of individual values from three independent experiments, each performed in duplicate (n = 6).

The coagulant activity of venom pools was evaluated as described by Valenzuela et al. [32] with modifications. Briefly, 40 μL of PBS and 50 μL of citrated human plasma were mixed in a flat-bottomed 96-well plate and prewarmed at 37°C for 15 min. To initiate plasma clotting, 50 μL of increasing dilutions of venom samples prewarmed to 37°C were added to the 96-well plate. Immediately after the addition of venom samples, absorbance was taken at 650 nm at 10-second intervals by a microtiter plate reader at 37°C (SpectraMax). We chose the clotting time as the time taken to reach a 0.04 absorbance value, where the increase in optical density (OD) is linear.

#### *In vivo* tests.

For *in vivo* tests, we used Balb/c mice weighing 18 – 20 g, following protocols approved by the Animal Ethical Use Committee of the Instituto Butantan (CEUA nº 4222230223, 21/June/2023). The procedures and animal species used in this study were chosen because the tests have already been standardized in mice, making the results with this species comparable to the data in the literature. The animals were housed in groups of five in 30 x 19 x 13 cm cages and were subjected to a 12-h light/12-h dark cycle. Their diet consisted of pelleted food and treated, filtered water ad libitum. The environment was maintained at a controlled temperature between 22°C and 25°C, with air conditioning and ammonia-safe exhaust systems. No intentional pain was inflicted on the animals, and the animal restraint procedure used causes a small amount of stress, performed for a short duration, thus avoiding the use of anesthetic drugs. Immediately after the conclusion of the experiments or upon observing suffering in the animals, they were euthanized by injection of ketamine hydrochloride (300 mg/kg) and xylazine hydrochloride (30 mg/kg). To minimize the number of animals in the study, we avoided tests as lethal doses of 50% of animals (LD50) or minimal hemorrhagic doses (MHD). Instead, we used data observed with a single fixed effective dose selected according to the literature and previous experiments performed at the lab.

The hemorrhagic activity was evaluated by the halo induced in the dorsal skin of groups of 5 mice injected intradermally (*i.d*.) with 50 µg venom in 50 µL of PBS. At three hours after the injection, the mice were euthanized by overdoses of anesthetic drugs; the skin of the dorsa was removed, and the hemorrhagic spots were measured (longest diameter multiplied by the diameter perpendicular to it). Results are expressed as mean ± SD of individual values of two independent experiments with five mice each (n = 10).

For the determination of myotoxic activity, 20 µg of venom samples diluted in 50 µL of PBS were injected intramuscularly (*i.m.*) into the gastrocnemius muscle of mice. After 3 hours, the blood was collected, and the sera were assayed for creatine kinase activity using a commercial kit (CK-UV, Bioclin) according to the manufacturer’s instructions. Groups of 5 animals were tested and compared to control groups, with animals injected only with PBS. Results are expressed as mean ± SD of individual values of two independent experiments with five mice each (n = 10).

The lethality induced by the venoms was evaluated by counting the live animals in groups of 5 mice at increasing times after venom injection, as previously described [33,34]. Groups of 5 mice were injected intraperitoneally (*i.p*.) with 50 µg venom/mouse in 50 µL of PBS. The number of surviving mice was recorded at 30, 90, 120, and 150 min and then at 4, 6, 24, and 48 h after the samples were inoculated, when the experiment was terminated. The data obtained were graphed on a survival curve plot in a linear scale up to 48 h and represent one of two independent experiments.

### Reactivity with antivenoms

We used in this study the antivenoms produced by Instituto Butantan (São Paulo, SP, Brazil), which consist of F(ab’)_2_ antibody fragments purified from the plasma of hyperimmunized horses with the following mixture of venoms: *Crotalus* antivenom (SAC) – batch 2301622/00, expire date September 2026, from horses immunized with venoms of *C. d. terrificus* (50%) and *C.d. collilineatus* (50%); *Bothrops* antivenom (SAB) – batch 2301634/00, expire date August 2026, from horses immunized with venoms of *Bothrops jararaca (*50%)*, B. alternatus* (12,5%)*, B. jararacussu* (12,5%)*, B. moojeni* (12,5%)*, and B. neuwiedi* (12,5%); and *Bothrops*-*Crotalus* antivenom (SABC) – batch 2301635/00, expire date September 2026, which is a mixture of equal parts both *Bothrops* and *Crotalus* antivenoms.

The reactivity of the antivenoms was evaluated by ELISA and Western blotting. Samples containing 100 µL whole venom (10 µg/mL) in carbonate buffer (pH 9.6) were used to coat Maxisorb microplates (Nunc). To determine the antibody titers, plates coated with whole venom were incubated with crescent dilutions of the antivenoms (from 1:10,000), followed by incubation with anti-horse IgG labeled with peroxidase (1:2,000). The reactions were developed using ortho-phenylenediamine and H_2_O_2_ as the enzyme substrate, and the products were detected at 490 nm. For antivenom titration, the reactions were performed in duplicate in two independent experiments, and the antibody titers were assumed as the maximal dilution resulting in OD values above 0.100. For Western blots, samples of crude venom (10 µg) were subjected to 12.5% sodium dodecyl sulfate-polyacrylamide gel electrophoresis (SDS-PAGE) under non-reducing conditions. After SDS-PAGE, the separated proteins were transferred to nitrocellulose membranes, which were then immersed in a blocking solution (5% non-fat milk in Tris-saline). The membranes were incubated with antivenoms (1:1,000) as the primary antibody and then with peroxidase-labeled goat anti-horse IgG (1:1,000). The reactive bands were detected by incubation with 4-chlor-α-naphthol and H_2_O_2_. Crotoxin, isolated from *C. d. terrificus* venom and Bathroxrhagin, isolated from *B. atrox* venom, were used as controls of antivenom reactivity with crotoxins and SVMPs, respectively. The results shown represent two independent experiments.

For neutralization assays, venom samples were incubated with *Bothrops* (SAB), *Crotalus* (SAC)*,* or *Bothrops*-*Crotalus* (SABC) antivenoms at ratios of 1 mL antivenom/1.5 mg venom, which is the proportion required of SAC to neutralize the reference venom (*C. d. terrificus*). The mixtures were incubated at 37°C for 30 minutes and then centrifuged at 2,000 × g for 15 minutes. The supernatants were used to assay the toxic or enzymatic venom activities described above. Venom samples incubated with PBS or injections of *Crotalus* antivenom only were used as a control.

### *In silico* tests

Sequences predicted by the *C. d. ruruima* venom gland transcriptome were aligned using Clustal W at https://www.genome.jp/tools-bin/clustalw with sequences with the highest BLAST hits [35] as follows: Cdr_PLA_01 aligned with crotoxin A of *C. d. terrificus* (P08878.1); Cdr_PLA_03 aligned with crotoxin B subunits of *C. d. terrificus* (Ba - P24027.1, Bb - P0CG56.1, Bd - P24027.1), crotoxin B subunit of *C. tzabcan* (Bc - A0A193CHJ5.1); Cdr_SVMPIII_01, and Cdr_SVMPIII_03 aligned with two PIII-class SVMPs from *C. d. durissus* (Q2QA02.1 and QIV647363), and bothropasin (O93523.2), a PIII-class SVMP isolated from *B. jararaca* venom. The 3D structure of Cdr_SVMPIII_01 and Cdr_SVMPIII_03 sequences were modelled by the I-TASSER package (https://www.rcsb.org/3d-view), with the predicted epitopes assessed according to the Epitope Prediction package from the Technical University of Denmark (DTU) Health Technology (https://services.healthtech.dtu.dk/) using 0.15 as epitope score.

### Statistical analysis

A two-tailed Student’s t-test with unequal sample variance was performed to evaluate differences between two paired groups. The analyses were performed using the software Microsoft Excel v.16.96.1 (2021-04-25).

## Results

### Intraspecific variability of *Crotalus durissus ruruima* venom

**Fig 1:**
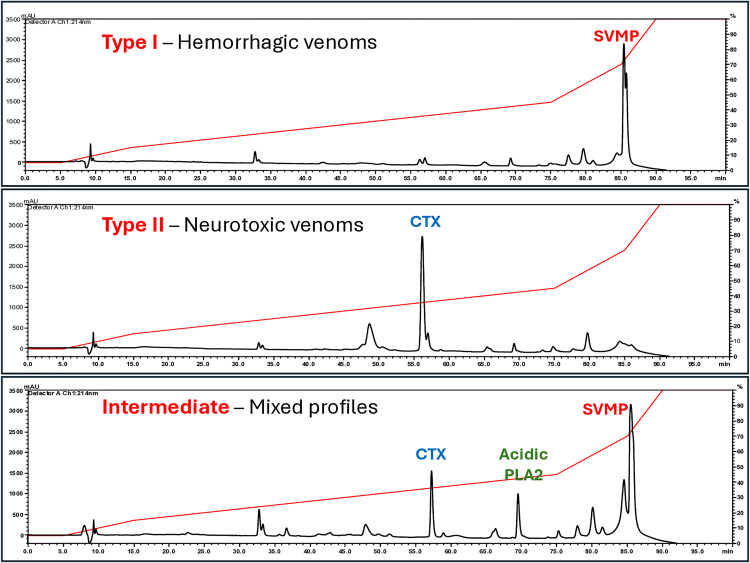
Representative chromatographic profiles of venom samples from *C. d. ruruima* fractionated by RP-HPLC: Individual venom samples (2 mg) were applied to a Phenomenex C-18 column. The mobile phases used were 0.1% TFA in water (solution A) or 0.1% TFA in acetonitrile (solution **B)**. Proteins were eluted in a gradient at 2 mL/min (5% B for 5 min, 5–15% B over 10 min, 15–45% B over 60 min, 45–70% B over 10 min, 70–100% over 5 min, and 100% B over 10 min). Separation was monitored at 214 nm. A – chromatogram representative of venoms CDR 4, CDR 9, CDR 13, and CDR 15; B – chromatogram representative of venoms CDR 1, CDR 2, CDR 3, CDR 5, CDR 6, CDR 7, CDR 10, CDR 14, SB 831, SB 833, and SB 1130; C – chromatogram representative of venoms CDR 8, CDR 11, CDR 12, and SB 834. The figure highlights the peaks that characteristically elute the basic subunit of Crotoxin (CTX), acidic forms of phospholipases A2 (acidic PLA2), and snake venom metalloproteinases (SVMP).

The variability in the composition of *C. d. ruruima* venom was studied using samples from 19 specimens collected primarily in the northeast region of Roraima State in Brazil. As illustrated in Table 1, out of the 19 samples, five were from juvenile snakes, 12 venom samples were yellow, and seven were white. Initially, the venoms were fractionated by reverse-phase chromatography using HPLC (RP-HPLC), and the main fractions of the chromatograms were characterized by mass spectrometry. We identified three distinct chromatographic profiles depicted in Fig 1: four venoms in which the highest peaks were eluted in the latter part of the chromatogram (> 80 min), in a peak that contained mostly snake venom metalloproteases (SVMPs), which are associated with Type I hemorrhagic crotalid venoms (Fig 1A); 11 venoms with the highest peaks eluted at approximately 55 min, characterized as the basic subunit of Crotoxin (CTX), typical of neurotoxic Type II venoms (Fig 1B); and four intermediate venoms, containing both CTX and SVMPs (Fig 1C). The five indicated peaks were collected from the representative chromatograms and subjected to MS/MS (S1 Table). SVMPs, the basic subunit of CTX, and acidic phospholipase A2 were identified as the predominant components, as shown in Fig 1. Individual chromatograms can be found in S1 Fig.

Venom samples were subsequently submitted to shotgun proteomic analyses with label-free identification and quantification of venom proteins based on a transcriptome assembled from data acquired from the venom glands of SB 0832, SB 0833, SB 0834, and SB 1130 snakes, annotated for venom toxin proteins. Forty-nine independent transcripts encoding venom toxins were fully sequenced. Based on this data, we successfully annotated four sequences corresponding to phospholipases A_2_ (PLA_2_s), three sequences of P-III class snake venom metalloproteinases (SVMPIIIs), four of P-II class SVMPs (SVMPII), thirteen sequences of snake venom serine proteinases (SVSP), fourteen of C-type lectin-like proteins (CTL), two of vascular endothelial growth factor (VEGF), and one sequence each of bradykinin potentiating peptide (BPP), cysteine-rich secretory protein (CRISP), ecto-5’-nucleotidase (NUC), L-amino acid oxidase (LAO), nerve growth factor (NGF), phosphodiesterase (PDE), hyaluronidase (HYAL), Kunitz inhibitors, and Warprin (S2 Fig).

**Fig 2:**
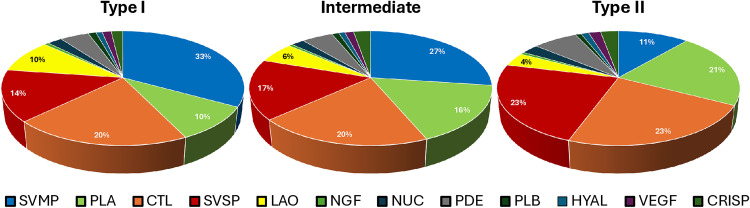
Distribution of toxin families in the proteomes of Type I, Type II, and intermediate type venoms of *C. d. ruruima*: Individual samples of *C. d. ruruima* venom were subjected to shotgun proteomic analysis, and the spectra were analyzed according to the master set annotated from the transcriptome data of *C. d. ruruima.* Pie charts represent the average composition of the venom from each phenotype, classified by toxin family. Relative expression was estimated by normalized total spectra counts (NTSC) by Scaffold 5.0.

The proteomes of individual venoms confirmed the existence of three phenotypes of *C. d. ruruima* concerning venom composition: Venoms exhibiting a higher abundance of specific isoforms of PIII-class SVMPs, classified as Type I; venoms with higher amounts of PLA_2_s designated as Type II; and venoms we referred to as intermediate, characterized by high expression of both PIII-class SVMPs and PLA_2_s (S2 Table). In Type I venoms, the average proportion of SVMPs constituted 33% of total toxins, while PLA_2_s made up only 10%. In contrast, Type II venoms displayed 11% SVMPs and 21% PLA_2_s. Furthermore, all venom phenotypes exhibited high levels of CTLs and SVSPs, contributing averages of 21% and 18%, respectively. LAO was also detected in every venom sample, averaging 6%. Small amounts of other venom components, such as VEGF, CRISP, NUC, NGF, PDE, PLB, and HYAL, were found in each sample. However, the venom proteomes did not detect the presence of BPPs, Kunitz inhibitors, or Warprin in any sample (Fig 2).

**Fig 3:**
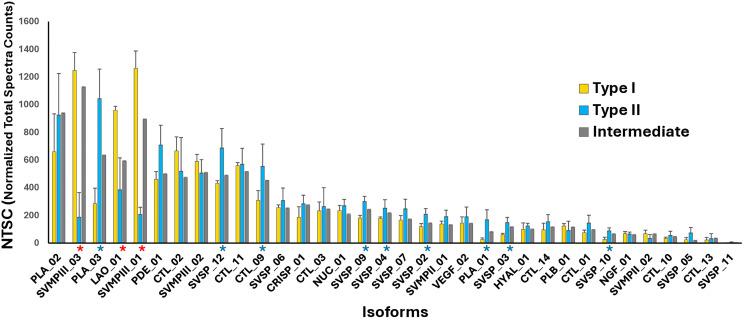
Average number of spectra of toxin isoforms in the venoms of *C. d. ruruima* from different phenotypes: Individual venom samples underwent shotgun proteomics analysis using the toxins master set annotated from the transcriptome data of *C. d. ruruima.* Bars represent the mean ± SD of normalized total spectra counts (NTSC) obtained via Scaffold 5.0 for each isoform in Type I (yellow bars, n = 4), Type II (blue bars, n = 11), or intermediate-type venoms (grey bars, n = 4). An asterisk (*) denotes the isoforms for which differences were statistically significant between groups of venoms (p < 0.01), indicating their predominance in Type I (*) or Type II (*) venom samples.

To better understand the venom phenotypes, the abundance of each toxin isoform was compared among the venoms classified as Type I, Type II, and intermediate. As shown in Fig 3, the most significant differences between Type I and Type II venoms lay in two isoforms of PIII-class SVMP (Cdr_SVMPIII_01 and 03) and two isoforms of PLA_2_s (Cdr_PLA_01 and 03), which predominated in Type I and Type II venoms, respectively. Additionally, the expression of Cdr_LAO_01 was higher in Type I venom, whereas the expression of some SVSPs was statistically higher in Type II venoms. The distribution of CTL was unrelated to the venom phenotype, although Cdr_CTL_09 was significantly more abundant in Type II venoms.

**Fig 4:**
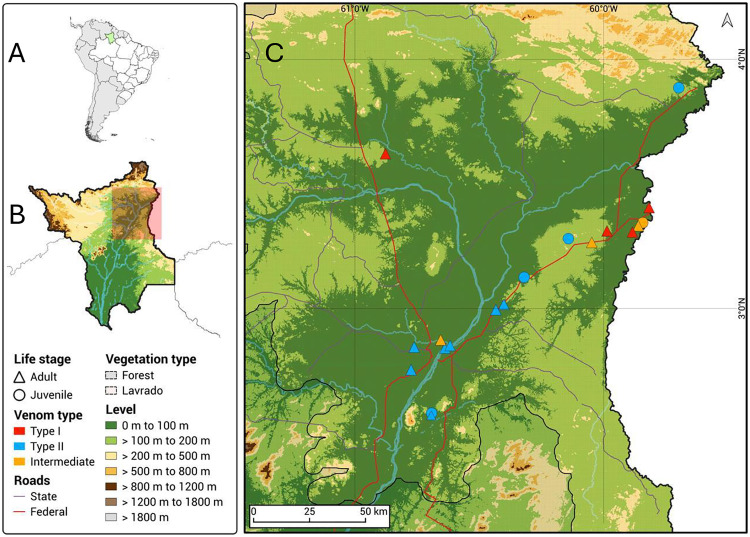
Geographical distribution of Type I, Type II, and intermediate venoms. An elevation map of the collection area illustrates the collection points of young individuals (circles) and adults (triangles) with venoms characterized as Type I (red), Type II (blue), or intermediate (orange) venoms. A: map of South America with Roraima State highlighted in green; B: map of Roraima State with the collection area highlighted as a pink square; C: collection area. Maps of geographical records were created using QGIS 3.34.4-Prizren, and the base layer of the map is available at https://ibge.gov.br/geociencias/downloads-geociencias.html.

Fig 4 summarizes the snake and venom characteristics observed in the samples used for this study.

To evaluate which factors could be driving the expression of the different phenotypes, we selected seven isoforms differentially expressed in Type I and Type II venoms (Cdr_SVMPIII_01 and 03, Cdr_PLA_01 and 03, Cdr_LAO_01, Cdr_SVSP_12, and Cdr_CTL_09) and analyzed them according to the snakes’ sex, ontogenetic stage, geographical location, and venom color.

**Fig 5:**
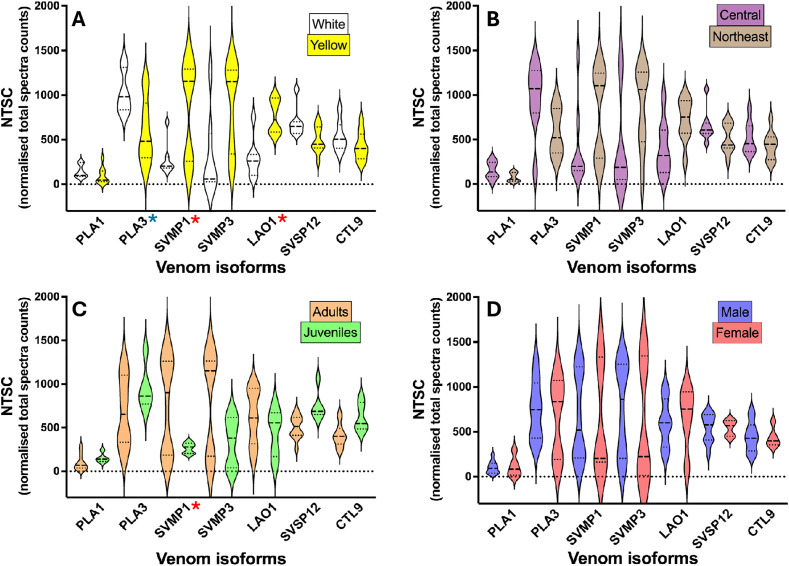
Comparison of violin plots for differentially expressed isoforms based on snake characteristics. The violins illustrate the distribution of normalized total spectra counts (NTSC) from the isoforms Cdr_SVMPIII_01 and 03, Cdr_PLA_01 and 03, Cdr_LAO_01, Cdr_SVSP_12, and Cdr_CTL_09 in samples categorized by venom color **(A)**, snake geographical location **(B)**, ontogenetic stage **(C)**, or sex **(D)**. An asterisk (*) indicates the isoforms for which differences were statistically significant between venom groups (p < 0.01), highlighting their predominance in Type I (*) or Type II (*) venom samples.

Fig 5 shows the violin plots comparing the expression of the selected isoforms in grouped samples. Most plots are bimodal, indicating the presence of both Type I and Type II phenotypes in each grouping. The yellow venoms demonstrated statistically significantly higher amounts of Cdr_SVMPIII_01 and lower quantities of Cdr_PLA_03 than white venoms and a significantly higher expression of LAO. Nevertheless, a group of venoms with elevated levels of Cdr_PLA_03 and reduced amounts of Cdr_SVMPIII_01 was also identified among the yellow venoms, suggesting that LAO expression and the color of the venom are not related to the distinction between Type I and Type II venom phenotypes (Fig 5A). Regarding the other comparisons, differences in the expression of selected isoforms were not statistically significant between snakes collected from the central or northeastern regions of Roraima State (Fig 5B), or male and female snakes (Fig 5D). However, concerning geographical location, in the graphs of snakes collected in central areas, it is notable that most samples are dispersed in a single cluster. In these samples, the expression of Cdr_PLA_03 is concentrated at higher levels, while Cdr_SVMPIII_01 and Cdr_SVMPIII_03 are at lower levels, suggesting that venoms from the central area tend to predominantly exhibit Type II phenotype. In contrast, in snakes from the northeast, the expression of the three isoforms was bimodal, equally distributed across lower and higher levels, indicating the presence of both Type I and Type II phenotypes in this region and explaining the lack of statistical significance (Fig 5B). Concerning the ontogenetic stage, it is evident from the graphs that adult snakes exhibit both venom phenotypes. However, venoms from juvenile snakes contain higher levels of Cdr_PLA_03 while Cdr_SVMPIII_01 and Cdr_SVMPIII_03 levels are lower, suggesting that the Type II phenotype may be dominant in juvenile snakes (Fig 4C). However, the data are statistically significant only for the Cdr_SVMPIII_01 isoform, which is insufficient to provide evidence of ontogenetic changes in *C. d. ruruima* venom (Fig 5C). Regarding the sex of the snakes, the violin plots did not reveal any aspects that could suggest differences in the expression of the selected isoforms related to sex. The bimodal plots may be associated with high and low expression of the isoforms that indicate Type I and Type II venoms in male or female snakes (Fig 5D). Regarding the other toxin families, there was no evidence of differences in Cdr_CTL_09 expression between the groups tested. However, the expression of Cdr_SVSP_12 was apparently higher in white venoms and venoms from juvenile snakes or adults collected from the central area, suggesting that SVSPs may predominate in Type II venoms. However, these differences are not statistically significant and, with the current data, cannot be correlated with venom biological activities.

### Structural characterization of the differentially expressed toxins

Cdr_PLA_001, Cdr_PLA_003, Cdr_SVMPIII_01, and Cdr_SVMPIII_03 are the isoforms differentially expressed in the venoms of Type I and Type II phenotypes. Thus, for their structural/functional characterization, we searched for homologous sequences already described in the venoms of Viper snakes using Blast search under the NCBI, using the “snakes” (Taxid:8570) subgroup of the UniprotKB/Swissprot databank.

As shown in Table 2, sequences with the highest identity with the differentially expressed isoforms correspond to homologous toxins from other *Crotalus* species. The sequence Cdr_PLA_01 aligned with 99% identity to the Crotoxin A subunit of *C. d. terrificus* and 97% identity with the A subunit of Mojave toxin, indicating that Cdr_PLA_01 corresponds to the acidic subunit of *C. d. ruruima* Crotoxin. Additionally, Cdr_PLA_03 corresponds to the B subunit of *C. d. ruruima* Crotoxin, as it aligned with 100% identity to the Crotoxin Bc subunit of *C. tzabcan*, and 99% identity with both the Crotoxin Bd and Bb subunits, as well as 94% identity with the Crotoxin Ba subunit from *C. d. terrificus*. Cdr_SVMPIII_03 is homologous to an SVMP described in *C. d. durissus* venom, demonstrating 99% identity. It is also homologous to SVMPs from *C. atrox* and *C. v. viridis* venom, with 98% and 97% identities, respectively. The similarity of Cdr_SVMPIII_03 with toxins from *Bothrops* venoms remains high at 88%, with substitutions expected due to the phylogenetic distance of the species. Interestingly, the database did not identify homologous sequences for the Cdr_SVMPIII_01 isoform. The highest identity for this isoform was a sequence from *C. d. terrificus* venom, not yet functionally characterised, with 91% identity, followed by SVMPs from *C. adamanteus, A. p. leucostoma, and B. jararaca* venoms with only 84%, 83%, and 66% identity, respectively.

**Table 2:** Sequences with the highest identity with the differentially expressed isoforms.

Toxin	Snake species	Accession number	Identity
Cdr_PLA_01			
Crotoxin A subunit	*Crotalus durissus terrificus*	P08878.1	99%
Mojave Toxin A subunit	*Crotalus scutulatus scutulatus*	P18998.2	97%
Cdr_PLA_03			
Crotoxin Bc subunit	*C. tzabcan*	A0A193CHJ5.1	100%
Crotoxin Bd subunit	*Crotalus durissus terrificus*	COHM14.1	99%
Crotoxin Bb subunit	*Crotalus durissus terrificus*	P0CG56.1	99%
Crotoxin Ba subunit	*Crotalus durissus terrificus*	P24027.1	94%
Cdr_SVMPIII_01			
SVMP 8	*Crotalus adamanteus*	J3SDW8.1	84%
SVMP AplVMPIII	*Agkistrodom pscivorus leucostoma*	C9E1S0.1	83%
SVMP VaH4-A	*Vipera ammodytes ammodytes*	V5TBK6.1	70%
PIII-class SVMP	*Crotalus durissus durisus*	Q2QA02.1	67%
SVMP Bothropasin	*Bothrops jararaca*	O93523.2	66%
Cdr_SVMPIII_03			
PIII-class SVMP	*Crotalus durissus durissus*	Q2QA02.1	99%
SVMP VAP2	*Crotalus atrox*	A4PBQ9.1	98%
SVMP VMPIII	*Crotalus viridis viridis*	C9E1R8.1	97%
SVMP Bothropasin	*Bothrops jararaca*	O93523.2	88%

**Fig 6:**
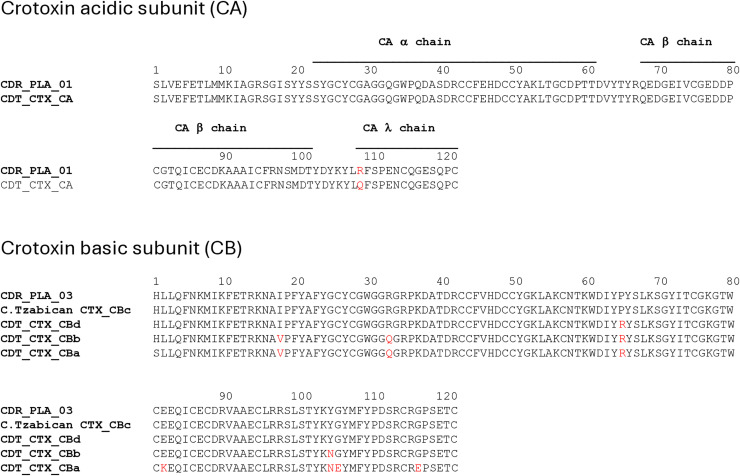
Alignment of and crotoxin subunits from *C. d. ruruima* venom: Crotoxin sequences Cdr_PLA_01 and Cdr_PLA_03 were aligned with the sequences of highest identity from venoms of *Crotalus spp* corresponding to crotoxin A subunit from *Crotalus durissus terrificus* (P08878.1); crotoxin Bc subunit from *Crotalus tzabcan* (A0A193CHJ5.1); crotoxin Bd subunit from *Crotalus durissus terrificus* (COHM14.1); crotoxin Bb subunit from *Crotalus durissus terrificus* (P0CG56.1); and crotoxin Ba subunit from *Crotalus durissus terrificus* (P24027.1) using the ClustalW program. The residues in red denote the substitutions compared to the *C. d. ruruima* sequence.

We then aligned the Crotoxin sequences, and we were able to verify that the CA subunit of the crotoxin from *C. d. ruruima* has a single substitution compared to the same subunit of Crotoxin from *C. d. terrificus*; however, the Q108R substitution is at the cleavage site of the **λ** chain of CA, and the consequence of this substitution is unclear. Substitutions were also observed in the α, β and γ chains of *C. d. terrificus* CA [36] and apparently do not interfere with Crotoxin function. The Crotoxin B subunit of *C. d. ruruima* was identical to the CBc subunit of *C. tzabcan*; a single substitution was observed compared to the CBd subunit, 4 for CBb and 7 for CBa from *C. d. terrificus* (Fig 6).

**Fig 7:**
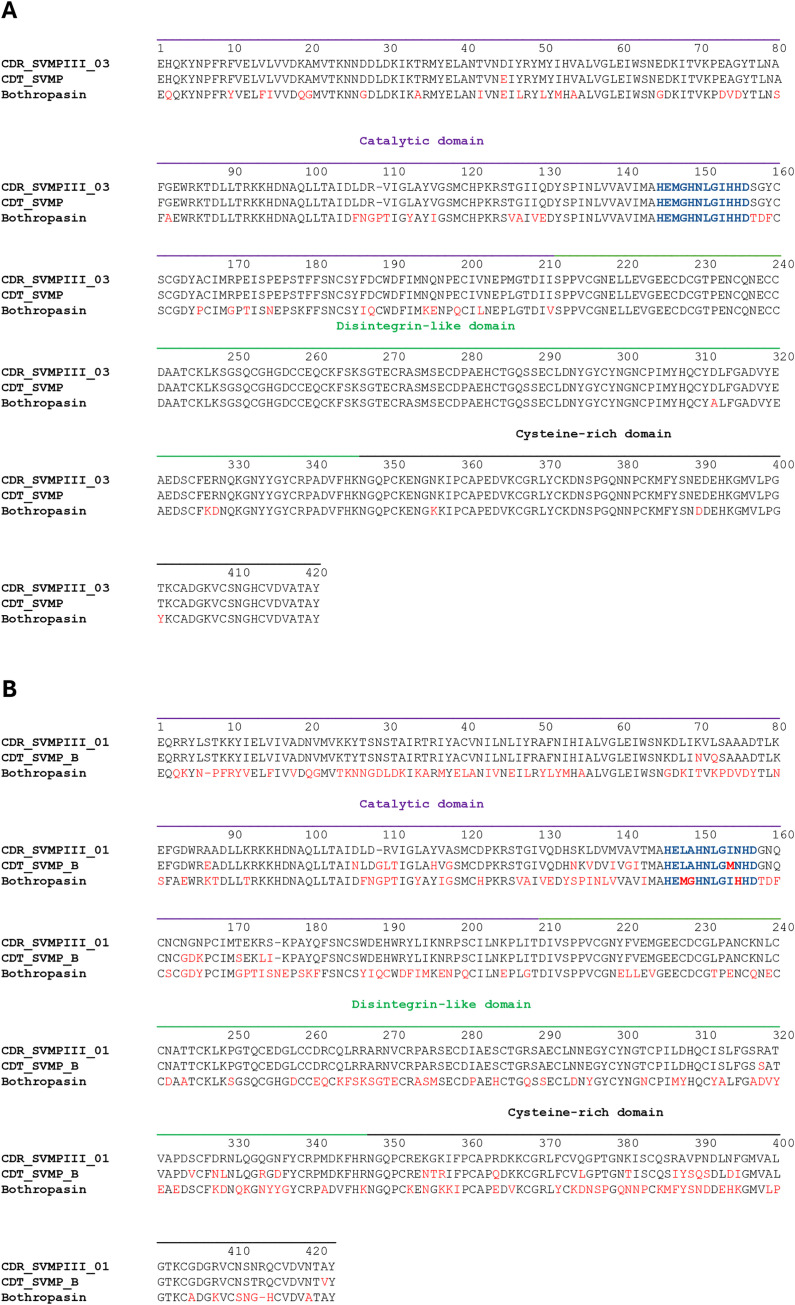
Alignment of SVMP sequences differentially expressed in *C. d. ruruima* venom: Cdr_SVMPIII_03 (A) and Cdr_SVMPIII_01 (B) sequences were aligned with a PIII-class SVMP from *Crotalus durissus durissus* (Q2QA02.1) and Bothropasin, a PIII-class SVMP from *Bothrops jararaca* (O93523.2) venoms using the ClustalW program. The residues in red denote the substitutions compared to the *C. d. ruruima* sequence and residues in blue correspond to the Zn-binding motif.

Different aspects have been observed in SVMP alignments. The Cdr_SVMPIII_03 sequence demonstrates a single substitution compared to the homologous SVMP from *C. d. collilineatus;* however, several substitutions were detected when compared to Bothropasin. The substitutions were located primarily in the catalytic domain region, which contains the catalytic characteristics of these enzymes, and in the cysteine-rich domain. Nevertheless, the zinc-binding motif is conserved, indicating that Cdr_SVMPIII_03 may hold multifunctional activities like Bothropasin (Fig 7A). Conversely, the Cdr_SVMPIII_01 sequence displayed multiple substitutions even when compared to the *C. d. terrificus* SVMP. The substitutions were identified throughout the sequence compared to Bothropasin, including within the disintegrin domain and the zinc-binding motif. Therefore, based on the primary structure, the biological activities of Cdr_SVMPIII_01 may differ from those exhibited by Bothropasin or other canonic SVMP and cannot yet be predicted due to the limited similarity of this sequence with those already characterized (Fig 7B).

### Functional activities of *Crotalus durissus ruruima* Type I and Type II venoms:

Differentially expressed toxins belong to the SVMP and Crotoxin families, responsible for the most toxic venom activities of *Crotalus* and *Bothrops* snakes, including lethality and disability observed in snakebite patients. Therefore, we subsequently compared the implications of the different phenotypes for the toxicity induced by *C. d. ruruima* venom. To achieve this*,* venom samples were pooled according to their phenotype, and the intermediate phenotype was not tested, attempting to minimize the number of animals required for *in vivo* tests with multiple samples. Thus, Type I venoms were categorized into Cdr-SVMP and Type II in Cdr-CTX pools, as detailed in Table 3.

**Table 3:** Venom classification according to the presence of SVMPs or CTX.

Pool	Venom type	Snake venoms included
**Cdr-SVMP**	Type I	Cdr 4, Cdr 9, Cdr 13, Cdr 15
**Cdr-CTX**	Type II	Cdr 1, Cdr 2, Cdr 3, Cdr 5, Cdr 6, Cdr 7, Cdr 10, Cdr 14, SB 831, SB 833, SB 1130
^*^	Intermediate	Cdr 8, Cdr 11, Cdr 12, SB 834

* Attempting to reduce the number of mice in experimental procedures, venoms from the intermediate phenotype were not pooled or submitted to functional characterisation.

**Fig 8:**
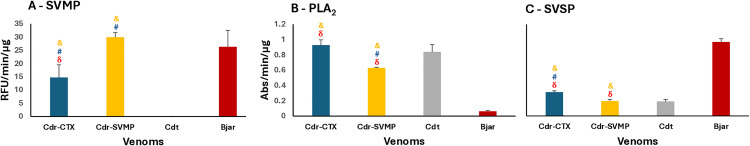
Enzymatic activities of the *C. d. ruruima* venom pools: venom samples from Cdr-SVMP, Cdr-CTX, *C. d. terrificus* (Cdt), and *B. jararaca* (Bjar) were subjected to the enzymatic assay of SVMPs through the hydrolysis of the FRET substrate (Abz-AGLA-EDDnp) (A), PLA2 activity through the hydrolysis of the chromogenic substrate (NOBA) (B), and SVSP by hydrolysis of the chromogenic synthetic substrate (L-BAPNA) (C). Readings obtained from wells containing only PBS were used as a reaction blank. The experiments were repeated three times in duplicate, and the data are represented as mean ± SD of individual tests (n = 6). Symbols denote significant difference (p < 0.05) related to *B. jararaca* venom (**δ**), *C. d. terrificus* venom (**#)**, and between Cdr-CTX and Cdr-SVMP (**&**).

The Cdr-SVMP and CTX venom pools were evaluated for enzymatic and toxic activities compared to the venoms of *C. d. terrificus* and *B. jararaca*, representatives of the *Crotalus* and *Bothrops* genera in Brazil, and the predominant antigens used in horse immunization to produce *Crotalus* and *Bothrops* antivenoms. As illustrated in Fig 8, the enzymatic SVMP and PLA_2_ activity values of the pools and controls confirmed the highest abundance of SVMPs or Crotoxin in Cdr-SVMP and Cdr-CTX pools, respectively. *C. d. terrificus* venom showed no SVMP activity in our tests. In contrast, both pools exhibited SVMP activity, which was significantly higher in Cdr-SVMP. No statistical significance was noted when comparing SVMP activity of Cdr-SVMP pool to the activity of *B. jararaca* venom (Fig 8A). Conversely, PLA_2_ activity was higher in the venom of *C. d. terrificus* and statistically similar to that of Cdr-CTX pool. In this instance, the PLA_2_ activity of Cdr-SVMP was significantly lower than that of Cdr-CTX and *C. d. terrificus* venom (Fig 8B). While examining SVSP activity, it was markedly higher in *B. jararaca* venom than in any sample of *Crotalus* venoms; SVSP activity was higher in Cdr-CTX than in Cdr-SVMP or *C. d. terrificus* venom (Fig 8C).

**Fig 9:**
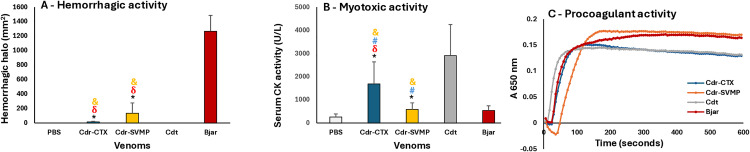
Functional activities of the *C. d. ruruima* venom pools: Venom samples from Cdr-SVMP, Cdr-CTX, *C. d. terrificus,* and *B. jararaca* were subjected to the hemorrhagic activity test by measuring the halo induced in the dorsal skin of mice three hours after the intradermal injection of 50 μg of the venoms (A); myotoxic activity by measuring creatine kinase levels in the serum of mice three hours after the injection of 20 μg of the venoms into the gastrocnemius muscle (B); and procoagulant activity, expressed as the increase in absorbance at 650 nm due to clot formation over five minutes, as indicated in the Methods section (C). A and B: the data represent the mean ± SD of two independent experiments involving five mice each (n = 10). Symbols denote significant difference (p < 0.05) related to PBS (*), *B. jararaca* venom (**δ**), *C. d. terrificus* venom (**#)**, and between Cdr-CTX and Cdr-SVMP (**&**). C: The plot represents a representative experiment out of three repetitions.

We also compared the Cdr-CTX and Cdr-SVMP pools of *C. d. ruruima* venom through *in vivo* tests that correspond to key functional activities of viper venoms (Fig 9). The hemorrhagic activity of both the Cdr-CTX and Cdr-SVMP pools was very low compared to the activity of *B. jararaca* venom, although it was significantly higher in the Cdr-SVMP pool than in the Cdr-CTX pool (Fig 9A). In contrast, the myotoxic activity of Cdr-CTX and Cdr-SVMP pools exceeded that of *B. jararaca* venom but remained lower than that of *C. d. terrificus* venom. The myotoxic activity of the Cdr-CTX pool seemed higher than that of Cdr-SVMP; however, this difference was not statistically significant (Fig 9B). The procoagulant activity of Cdr-CTX was comparable to that of *C. d. terrificus* and *B. jararaca* venoms (clotting times 37.0 ± 1.1, 37.0 ± 0.9, 33.0 ± 1.7 seconds, respectively), whereas the activity of the Cdr-SVMP pool (clotting time 65.0 ± 3.0 seconds) was significantly lower than that of the other venom samples (p < 0.01) (Fig 9C).

**Fig 10:**
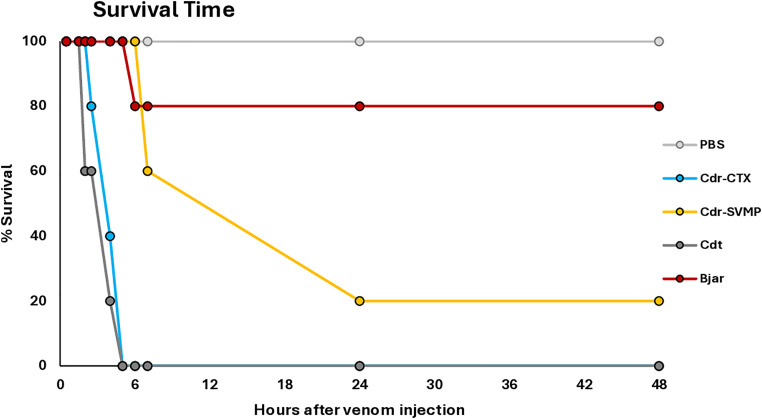
Survival time of mice injected with *C. d. ruruima* venom pools: Groups of five mice were injected *ip* with 50 μg/mouse of Cdr-SVMP, Cdr-CTX, *C. d. terrificus* (Cdt), or *B. jararaca* (Bjar) venom pools, and the number of survived mice was observed at 30, 90, 120, and 150 min and then at 4, 6, 24, and 48 h. The result is representative of two independent experiments.

Next, we evaluated the lethality of the venom samples by recording the survival time of mice injected intraperitoneally with 50 μg of Cdr-SVMP, Cdr-CTX, *C. d. terrificus,* or *B. jararaca* venom pools. As shown in Fig 10, the lethality in mice injected with Cdr-CTX or *C. d. terrificus* venom was observed between 60 and 90 minutes, with the animals not surviving longer than five hours. Conversely, all mice injected with the Cdr-SVMP pool survived for six hours; two died before seven hours, and two before 24 hours. One mouse injected with *B. jararaca* venom died between six and seven hours, while the other four survived beyond 48 hours, the maximum observation time (Fig 10).

### Neutralization of *C. d. ruruima* venom activities by antivenoms

The antivenom produced in Brazil to treat accidents by *Crotalus* snakes is prepared from the plasma of horses hyperimmunized with venoms of *C. d. terrificus* (50%) and *C.d. collilineatus* (50%). Venoms of these two species are rich in crotoxin but contain small amounts of SVMPs [37], which could compromise the efficacy of the *Crotalus* antivenom against accidents involving *C. d. ruruima* presenting Type I venom phenotype. Conversely, the *Bothrops* antivenom is prepared through immunization with 50% *Bothrops jararaca,* 12.5% *B. alternatus,* 12.5% *B. jararacussu,* 12.5% *B. moojeni, and* 12.5% *B. neuwiedi* venoms, all of which are rich in SVMPs and could serve as an alternative for the treatment of Type I *C. d. ruruima* envenomings. Considering that venoms with type II phenotype have major toxins identical to *C. d. terrificus* Crotoxin, its neutralisation by the *Crotalus* antivenom is assured. However, it remains unclear whether *Crotalus* or *Bothrops* antivenoms could neutralize Type I venoms. Therefore, our next step was to evaluate *in silico, in vitro,* and *in vivo* the reactivity and neutralisation of *C. d. ruruima* venom of both phenotypes by *Crotalus*, *Bothrops*, or *Bothrops*-*Crotalus* antivenom, which is a commercially available mixture of both.

**Fig 11:**
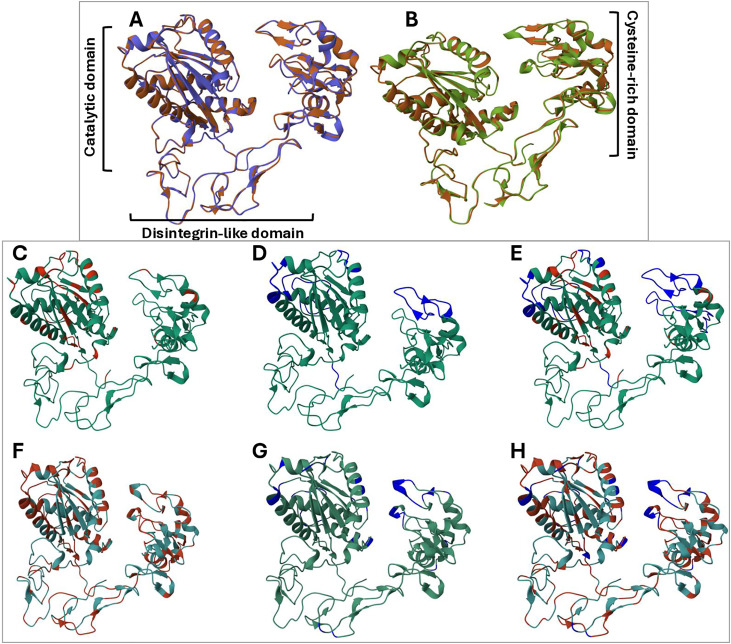
Molecular modelling and epitope prediction of Cdr_SVMPIII_03 and Cdr_SVMPIII_01 sequences: Three-dimensional structures of Cdr_SVMPIIIs were modelled using the I-TASSER package (https://www.rcsb.org/3d-view), and the predicted epitopes were obtained from the Epitope Prediction package provided by DTU (https://services.healthtech.dtu.dk/). Illustrations depict the Cdr_SVMPIII_03 (A, C, D, E) and Cdr_SVMPIII_01 (B, F, G, H) structures overlapping with the Bothropasin structure (3dslA.pdb) **(A, B)**. Regions with amino acid substitutions compared to Bothropasin are highlighted in red **(C, F)**, epitopes predicted for reactivity with antibodies are highlighted in blue **(D, G)**, and merged substitutions and epitopes are shown **(E, H)**.

We first modelled the 3D structure of the Cdr_SVMPIII_01 and Cdr_SVMPIII_03 sequences using the I-Tasser package [38,39]. The best template for both structures was the Bothropasin structure (3dslA) with TM-Scores of 0.982 and 0.987, respectively. Fig 11A shows the structure of Cdr_SVMPIII_03 overlapped with Bothropasin, and Fig 11B shows the same overlap with the structure of Cdr_SVMPIII_01. The general structures of both Cdr_SVMPs overlapped perfectly with Bothropasin, a canonical structure of PIII-class SVMP [40]. However, according to the sequence alignments, several substitutions were noted between Cdr_SVMPs and Bothropasin. Thus, we marked the amino acid substitutions in each SVMP structure compared to Bothropasin (Fig 11C and 11F). Furthermore, we identified the predicted epitopes that react with B-cell receptors in the same structures (Fig 11D and 11G). When merging the structures (Fig 11E and 11H), we observed in Cdr_SVMPIII_03 (Fig 11E) that some locations of amino acid substitutions found in the α-helix of the catalytic and cysteine-rich domains overlap with several of the predicted epitopes and, consequently, may interfere with the interaction with *Bothrops* antivenom. Additionally, Cdr_SVMPIII_01 exhibited numerous amino acid substitutions compared to Bothropasin across the different domains (Fig 11F), overlapping most regions with the putative B-cell epitopes (Fig 11G and 11H). Thus, it is improbable that Cdr_SVMPIII_01 could be recognized and/or neutralized by antibodies raised against Bothropasin-like SVMPs, and it is also unlikely that *Bothrops* antivenom could neutralize the enzymatic activity and binding to ECM proteins of the protease.

Next, we evaluated the reactivity of the antivenoms with the two venom phenotypes of *C. d. ruruima* species. As shown in Table 4, the *Crotalus* antivenom reacted with the Cdr-SVMP and Cdr-CTX pools at identical antibody titers, similar to those that reacted with *C. d. terrificus*. As expected, the *Bothrops* antivenom preferentially recognized *B. jararaca* venom, while the *Bothrops*-*Crotalus* antivenom reacted at higher titers with all venom samples.

**Table 4:** ELISA antibody titres* of commercial antivenoms against *C. d. ruruima* venom pools.

	Antivenoms		
	*Bothrops*	*Crotalus*	*Bothrops-Crotalus*
**Cdr-SVMP**	512	2,048	4,096
**Cdr-CTX**	512	2,048	4,096
***C. d. terrificus***	128	2,048	2,048
***B. jararaca***	2,048	256	2,048

*Antibody titers were defined as the maximal dilution resulting in an OD above 0,100.

**Fig 12:**
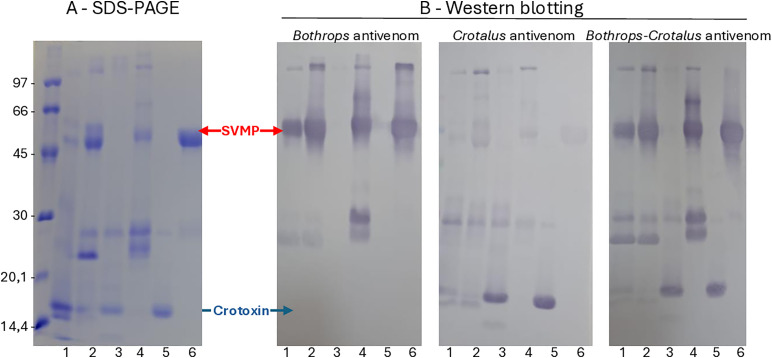
Reactivity of antigens from *C. d. ruruima* venom pools with antivenoms produced at the Butantan Institute. Venom samples of Cdr-CTX (1), Cdr-SVMP (2), *C. d. terrificus* (3), *B. jararaca* (4), crotoxin, isolated from *C. d. terrificus* venom (5), and bathroxrhagin, an SVMP isolated from *B. atrox* venom (6), were fractionated by SDS-PAGE on a 12.5% acrylamide gel under non-reducing conditions and stained with Coomassie blue (A) or transferred to nitrocellulose membranes, which were then incubated with *Bothrops*, *Crotalus*, or *Bothrops*-*Crotalus* antivenoms as primary antibodies, followed by incubation with peroxidase-labelled anti-horse IgG. Reactive bands were detected by incubation with 4-chloro-a-naphthol and H_2_O_2_ **(B)**. The numbers on the left indicate the mobility of the molecular mass markers in kDa.

Despite the high antibody titers of SAC against the venom of the Type I phenotype, it did not recognize SVMP bands in Western blots, reacting mainly with the Crotoxin bands on venoms of both phenotypes (Fig 12). On the other hand, SAB had low antibody titers against both *C. d. ruruima* pools but strongly recognized SVMP bands in Western blots. SABC was the most efficient in identifying the prominent bands of both venom phenotypes.

**Fig 13:**
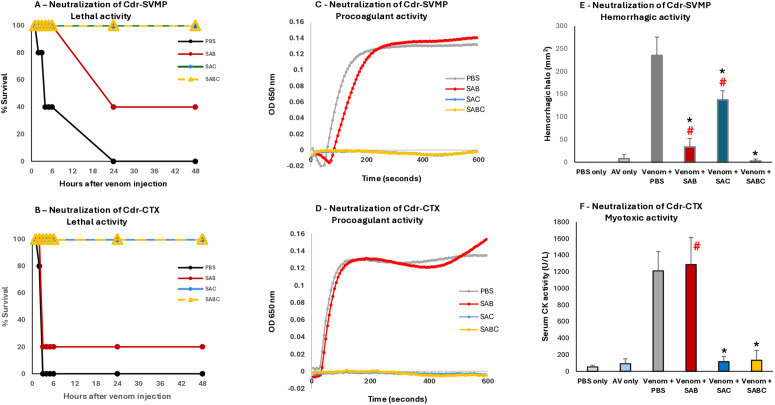
Neutralization of the toxic activities of *C. d. ruruima* venom pools by antivenoms. Venom samples of Cdr-SVMP (A, C, E) and Cdr-CTX (B, D, F) were incubated with *Bothrops* (SAB), *Crotalus* (SAC), *Bothrops*-*Crotalus* (SABC) antivenoms or with PBS (control) for 30 minutes at 37 °C, then centrifuged. The supernatants were tested to determine the survival time (A, B) as well as the residual procoagulant **(C, D)**, hemorrhagic **(E)**, and myotoxic (F) activities as described in the Methods. A, B, C, and D: the graphs are representative of two independent experiments; E and F: the data represent the mean ± SD of two independent experiments involving five mice each (n = 10). Symbols denote significant difference (p < 0.05) related to positive control PBS/venom group (*), or negative control antivenom only group (**#)**.

Next, we evaluated the neutralization of the toxic activities of *C. d. ruruima* venom by SAB, SAC, and SABC antivenoms (Fig 13).

The results of these assays indicated that *Crotalus* antivenom completely neutralized the toxic activities, such as lethality and procoagulant activity, of both Cdr-SVMP and Cdr-CTX venom pools, as well as the myotoxic activity of the Cdr-CTX pool (Fig 13). However, SAC partially neutralized the Cdr-SVMP hemorrhagic activity, which was neutralized by *Bothrops* antivenom (Fig 13E). Interestingly, *Bothrops* antivenom had a minimal effect on the neutralization of the lethality and a very little impact on the neutralization of the procoagulant activity of both Cdr-SVMP and Cdr-CTX venom pools (Fig 13A, 13B, 13C and 13D red lines). In the same experiments, *Bothrops* antivenom completely neutralized lethality and the procoagulant activity of *B. jararaca* venom, which was used as a control (S3 Fig). This evidence suggests that the epitopes shared between SVMPs from the *C. d. ruruima* Cdr-SVMP Type I phenotype and *B. jararaca* venom are sufficient for antibody recognition in *in vitro* reactions, as observed in the Western blots; however, epitopes disrupted by sequence substitutions are likely to play more significant roles in the toxic activity of the toxins, except for the hemorrhagic activity. Consistent with this, *Bothrops* antivenom partially reduced the SVMP enzymatic activity of the Cdr-SVMP venom pool, indicating that *Bothrops* antivenom may not be recommended for *C. d. ruruima* envenomings. Despite this, the combination of SAB and SAC in the *Bothrops*-*Crotalus* antivenom (SABC) proved very effective, as SABC completely neutralized all tested activities of both Cdr-SVMP and Cdr-CTX venom phenotypes of *C. d. ruruima* (Fig 13).

## Discussion

This manuscript describes a detailed examination of 19 individual venom samples, comprehensively characterizing the *C. d. ruruima* venom dichotomy based on the structural characterization of isoforms differentially expressed in Type I or Type II venoms, the biological activities of each phenotype, and the implications for treating snakebites in Roraima State of Brazil. Our data indicate that Type I venoms are characterized by the differentially higher expression of two isoforms of PIII-class SVMPs: Cdr_SVMPIII_01 and Cdr_SVMPIII_03, which may be associated with the higher proteolytic activity towards SVMP substrates and the hemorrhagic activity, which, although very low, was detectable only in the Type I Cdr-SVMP venom pool. The expression of LAO was also significantly higher in Type I venoms, thereby explaining the previous data linking venom color with hemorrhagic venoms. However, LAO was also found in yellow samples of Type II venoms, indicating that venom color cannot be regarded as a discriminator of the distinct venom phenotypes. In contrast, venoms classified as Type II exhibited a marked expression of Crotoxin subunits, associated with increased phospholipase A_2_ and myotoxic activities, leading to the heightened lethality of Type II Cdr-CTX venom pool. A statistically significant increase in SVSP expression was also observed in Type II venoms, resulting in a faster clotting time for Cdr-CTX than Cdr-SVMP venom pools. However, the differences in SVSP expression between Type I and Type II venoms were not as marked as those for SVMPs or Crotoxin.

*C. d. Ruruima exhibits* both Type I and Type II venom phenotypes, and SVMPs or Crotoxin appear to be selected for in these venoms. However, selection does not operate very strictly in this species, as the differentially expressed isoforms appear in both venom types, albeit in markedly different concentrations. Moreover, some venoms include nearly equal amounts of SVMPs and Crotoxin and are classified as intermediate. When analyzing *C. sucutulatus*, intermediate venoms have also been identified in individuals that occur in regions of inferred introgression. In *C. scutulatus*, mixed phenotypes were comparatively rare, supporting the fixation of alternative venom phenotypes on a fine geographic scale [13]. However, in *C. d. ruruima*, intermediate venoms are found in 4 out of 19 specimens distributed across the central and northeastern regions, evidencing the balance of both phenotypes within the species.

Ontogenetic shifts between Type I and Type II venom phenotypes have also been described for rattlesnakes such as *Crotalus viridis oreganus* [14], Central American *C. durissus* [19], *C. cumanensis* from Venezuela [18], and lineages of *C. molossus* from Mexico [15]. In these species, the venoms of newborn specimens exhibit significantly higher lethal and myotoxic activities, with weak proteolytic properties, in contrast to the venoms of adult specimens, which have a low concentration of crotoxin and higher protease activity. Thus, Type II venoms were characterized as the juvenile phenotype. In *C. d. terrificus* snakes, the expression of Type II venoms throughout their lives has been correlated to an adaptive paedomorphic trait driven by the gain of neurotoxicity to rodents along the north-south axis of *Crotalus* radiation in South America [17]. In *C. d. ruruima*, the venoms of juvenile snakes exhibited higher concentrations of Crotoxin and lower amounts of SVMPs, which could be attributed to Type II venoms as a characteristic of juvenile snakes. Nevertheless, conservation of the paedomorphic trait was not observed. Both phenotypes were found in adult snakes, providing further evidence that balancing selection tends to maintain both phenotypes within the species, offering a selective advantage over the conservation of the paedomorphic trait.

Type I and Type II phenotypes of *Crotalus* snakes have been correlated with distinct locations throughout the species’ distribution [12,41,42]. Although the data were not statistically significant, we noted a tendency towards Type II in the venoms of *C. d. ruruima* snakes collected from the central/southern area of Roraima compared to the Type I venoms gathered in the northeast. Rattlesnakes from Central America exhibit Type I venoms in their adult stage. In contrast, in South America, rattlesnakes display Type II venoms in their adult stage [17,19]. Thus, it is tempting to suggest that in the northern part of South America, an intergradation of Type I and Type II venom phenotypes may be occurring, which could explain the higher concentration of Type II venoms in the southern region and the mixed profiles in the northern parts of Roraima State. Supporting this hypothesis, intraspecific variability in the venoms of *C. d. durissus* and *C. d. cumanensis* from Colombia and Venezuela display variability in their composition and include the Type I phenotype in adult snakes [18,21,43].

The mechanisms by which this venom variation arises are not fully understood, and various underlying hypotheses have been proposed. Dowell and collaborators reported strong evidence that the expression of Type I and Type II venom phenotypes in North American rattlesnakes is associated with recent lineage-specific gene loss within the PLA_2_ or SVMP multigene clusters, suggesting that the genomic presence or absence of members of the SVMP and PLA_2_ gene families determines venom phenotype [44,45]. However, the presence or absence of these genes explains some, but not all, of the variation among venom types. The ontogenetic shifts between Type II and Type I phenotypes provide evidence that regulatory mechanisms may also explain venom variation. Durban and collaborators demonstrated age-dependent changes in the concentration of miRNAs that could modulate the expression of SVMP or Crotoxin transcripts [46]. Moreover, there was evidence of intermediate venoms expressing SVMPs and Crotoxin in *C. s. scutulatus* [12] and in *C. d. ruruima* (this paper), which further supports that these toxins can be expressed simultaneously, and the level of expression of these genes may relate to post-transcriptional modulation. Recently, Hogan and collaborators published a robust paper showing that ontogenetic expression changes in the venoms of *Crotalus adamanteus* were significantly correlated with epigenomic changes within gene promoters or enhancers and the up-regulation of transcription factors in adults [47]. Considering this evidence, the mechanisms responsible for venom variation may reside at the genomic, transcriptional, and post-transcriptional levels, and further studies may shed more light on the control of such an essential trait for snake adaptability.

The dichotomy of *C. d. ruruima* venom poses significant consequences for public health in Brazil. Since rattlesnake venoms in Brazil are predominantly Type II, the antivenom produced in the country to treat *Crotalus* snakebites is derived from the plasma of horses immunised with *C. d. terrificus* and *C. d. collilineatus*, both Type II venoms with very low concentrations of SVMPs. This raises a serious question regarding the efficiency of this antivenom in treating patients affected by Type I *C. d. ruruima* venoms. Some authors have observed local effects on some patients bitten by rattlesnakes in Roraima and suggested that *Bothrops* antivenom could be effective for this group [2,48,49]. The rationale is that the *Bothrops* antivenom comes from the plasma of horses immunized with venoms from *Bothrops* snakes, which are rich in SVMPs [50]. Indeed, 50% of the immunizing mixture comprises the venom from *Bothrops jararaca*, from which Bothropasin, a PIII-class SVMP, is the major antigen [51]. Thus, we compared the structure of the SVMPs upregulated in Type I venoms to Bothropasin and the biological activities of Type I *C. d. ruruima* venom with those of *B. jararaca* venom. The PIII-class SVMPs differentially expressed in Type I venoms exhibited 66% and 88% identity with Bothropasin for Cdr_SVMPIII_01 and Cdr_SVMPIII_03, respectively. The modelled 3D structure of both sequences overlapped with the Bothropasin template; however, several amino acid substitutions were observed and analyzed concerning biological activity and reactivity with antivenoms. The key residues in the zinc-binding motif were conserved in Cdr_SVMPIII_01, Cdr_SVMPIII_03, and Bothropasin, conferring high catalytic activity towards metalloproteinase substrates, similar in *C. d. ruruima* subtypes and *B. jararaca* venoms. However, substitutions in the cysteine-rich domain in both Cdr_SVMPIII_01 and Cdr_SVMPIII_03 and even in the disintegrin-like domain at Cdr_SVMPIII_01 have been noticed. These non-catalytic domains have been correlated with binding to extracellular matrix proteins [52–54] and comprise the epitopes reactive to monoclonal neutralizing antibodies [55,56]. Thus, substitutions in the non-catalytic domains may be responsible for the lower hemorrhagic activity of Type I *C. d. ruruima* venom compared to *B. jararaca* venom. On the other hand, Type I venom displayed more significant toxicity than *B. jararaca* venom, with higher lethality in rodents. Two hypotheses may arise from these observations: first, other basal components such as CTLs, SVSPs, basic PLA_2_s, or PII-class SVMPs, shared to both Type I and Type II venoms, may configure a highly toxic background for Type I venoms; second, the numerous amino acid substitutions in Cdr_SVMPIII_01, differentially expressed in Type I venom, may have resulted in an enzyme with a distinct mechanism of action that exhibits more significant toxicity, not seen in *B. jararaca* venom.

The reactivity with antivenoms confirmed the hypothesis stated above. *Bothrops* antivenom recognized PIII-class SVMP bands in Western blots; however, it reacted similarly with Type I and Type II venoms, displaying lower antibody titers than those of *Crotalus* antivenom reacting against the same samples. Regarding the neutralization by antivenoms, it is essential to note that our study faced some limitations related to the *in vivo* experimental conditions. To comply with ethical standards for animal use, we minimized the number of animals by choosing a single effective dose based on the literature and previous laboratory experiments with venom and antivenom. This approach avoided tests that involve large numbers of animals, such as lethal doses for 50% of the animals (LD50), minimal haemorrhagic doses (MHD), or the median effective dose (ED50) of antivenoms. A detailed quantitative comparison of antivenom potency was not the goal of this study and will be addressed in a follow-up study. Nevertheless, *Crotalus* antivenom completely neutralized the myotoxic, pro-coagulant, and lethal activities of Type I and Type II venoms. Still, it partially neutralized the hemorrhagic activity of Type I venom. In contrast, *Bothrops* antivenom was effective only in neutralizing the hemorrhagic activity of Type I venom, with minimal effect on neutralizing the lethality and pro-coagulant activity of both Type I and Type II *C. d. ruruima* venoms. A similar result was obtained by Muniz and collaborators, in which *Crotalus* antivenom neutralized all *C. d. ruruima* venom activities, except for the hemorrhagic activity of Type I venom samples [49]. Nevertheless, the efficacy of *Bothrops* antivenom in neutralizing venom-induced hemorrhage may not go unnoticed. Although the *Crotalus* antivenom was effective in neutralizing the lethal, myotoxic, and *in vitro* coagulant activities of the venoms of *C. d. terrificus*, *C. simus*, and *C. d. cumanensis* [18], as well as the lethal effect of *C. d. ruruima* [24] and *C. basiliscus* venoms [57], this antivenom was ineffective in neutralizing the hemorrhagic activity of *C. d. ruruima* venom [24,49], as observed in this study.

Therefore, it has been attributed to treating rattlesnake bites in Roraima State using *Bothrops*-*Crotalus* antivenom. Indeed, as demonstrated here, the mixture of *Crotalus* with *Bothrops* antivenoms completely neutralized all tested activities of both phenotypes of *C. d. ruruima*. However, there is little evidence regarding the advantages of using *Bothrops-Crotalus* antivenom rather than *Crotalus* antivenom in treating *C. d. ruruima* snakebite patients. A clinical study was reported involving patients from Bonfim [48], the site where we noticed the highest incidence of Type I venoms. Among the symptomatic patients, 70.3% exhibited systemic manifestations such as blurred vision, myalgias, myasthenic facies, palpebral ptosis, muscle weakness, and headache, all characteristic of envenomation by *Crotalus* with Type II venoms, and they received *Crotalus* antivenom. Nine patients exhibited local pain and edema, and were suspected of envenoming by *Bothrops*, and were treated with *Bothrops* antivenom. Despite the weak neurological symptoms, these patients evolved to changes in muscle enzymes and were treated additionally with *Crotalus* antivenom. However, the study lacked an unequivocal identification of the offending snake or tests confirming that the incidents were by Type I *C. d. ruruima.* In another clinical study, the follow-up of four patients envenomed by *C. d. ruruima* has shown that neurotoxic signs ceased, and hemostasis parameters and CK values returned to normal 24 hours after treatment with the *Bothrops-Crotalus* antivenom. The efficacy outcome was defined as the suppression of coagulopathy, improvement of creatine phosphokinase activity, and suppression of neurological manifestations within the first 24 hours of follow-up [58]. Still, once more, there was no evidence that this study included envenoming with Type I specimens. Moreover, as shown here, the *Crotalus* antivenom neutralizes coagulopathy, kidney injuries, or neurological manifestations in *Crotalus* accidents. Therefore, the advantage of using the *Bothrops-Crotalus* antivenom in these patients remains unclear.

Thus, the use of *Bothrops* antivenom in incidents involving *C. d. ruruima* snakes of the Type I phenotype rely solely on experimental evidence. Furthermore, it is important to acknowledge that antivenom treatments entail the administration of a substantial amount of heterologous immunoglobulins to patients and should be limited to the most specific immunoglobulins possible. In *Bothrops-Crotalus* antivenom, the immunoglobulins derived from *Crotalus* immunization would sufficiently neutralize most venom toxicity, including lethality, hemostatic issues, and kidney injuries. In contrast, immunoglobulins from the *Bothrops* antivenom would be necessary to counteract only the hemorrhagic effect, which, as demonstrated here, is not a severe toxicity even for Type I venoms. In this sense, to support the distribution and administration of *Bothrops*-*Crotalus* antivenom in Roraima, a clinical trial is urgently needed to assess the comparative efficacy of *Bothrops*-*Crotalus* or only *Crotalus* antivenoms. Meanwhile, the *Bothrops*-*Crotalus* antivenom could be administered, although it is essential to be aware of the limited effectiveness of *Bothrops* antibodies in treating *Crotalus* snakebite patients.

## Supporting information

S1 Fig RP-HPLC chromatographic profiles of individual *Crotalus durissus ruruima* snake venoms. Individual venom samples (2 mg) were applied to a Phenomenex C-18 column. The mobile phases used were 0.1% TFA in water (solution A) or 0.1% TFA in acetonitrile (solution B). Proteins were eluted in a gradient at 2 mL/min (5% B for 5 min, 5–15% B over 10 min, 15–45% B over 60 min, 45–70% B over 10 min, 70–100% over 5 min, and 100% B over 10 min). Separation was monitored at 214 nm.
(S1_Fig.PDF)

S2 Fig Master Seq of *Crotalus durissus ruruima* venom gland toxins. Annotation of the protein sequences predicted from the transcriptome of venom glands from SB831, SB833, SB834, and SB1130 specimens.
(S2_Fig.PDF)

S3 Fig Neutralisation of procoagulant activities of *C. d. ruruima* venom pools, and *C. d. terrificus* and *B. jararaca* venoms by antivenoms. Venom samples of Cdr-SVMP (A) and Cdr-C TX (B), C. d. terrificus venom (C), and B. jararaca venom (D) were incubated with Bothrops (SAB), Crotalus (SAC), Bothrops-Crotalus (SABC) antivenoms or with PBS (control) for 30 minutes at 37 °C, then centrifuged. The supernatants were tested to determine the residual procoagulant activity as described in the Methods.
(S3_Fig.TIF)

S1 Table Quantification of protein families expressed in the venom samples. MSFragger was set up to search a reverse concatenated in-house database consisting of the protein sequences predicted from a non-redundant master set containing venom toxins annotated from the transcriptome of venom glands from SB831, SB833, SB834, and SB1130 specimens, assuming the digestion by trypsin. The Normalized Total Spectra Counting (NTSC) was used to estimate the abundance of the toxin groups.
(S1_Table.XLSX)

S2 Table Quantification of isoforms expressed in the venom samples. MSFragger was set up to search a reverse concatenated in-house database consisting of the protein sequences predicted from a non-redundant master set containing venom toxins annotated from the transcriptome of venom glands from SB831, SB833, SB834, and SB1130 specimens, assuming the digestion by trypsin. The Normalized Total Spectra Counting (NTSC) was used to estimate the abundance of independent proteins and toxin groups.
(S2_Table.XLSX)

S1 Raw Data The raw data from quantitative experiments are presented as an Excel file, with each spreadsheet corresponding to the data for the respective figure in the paper. (S1_Raw_Data.XLSX)
