# Supplementary material for: Phenotypic dichotomy in Crotalus durissus ruruima venom and potential consequences for clinical management of snakebite envenomations
Source: PLoS Negl Trop Dis. 2025 Aug 1;19(8):e0013296. doi: 10.1371/journal.pntd.0013296 (PMC12327661; doi:10.1371/journal.pntd.0013296)

Supplementary Figure 01: RP-HPLC chromatographic profiles of individual *Crotalus durissus ruruima* snake venoms. mobile phases used were 0.1% TFA in water (solution A) or 0.1% TFA in acetonitrile (solution B). Proteins were eluted in a gradient at 2 mL/min (5% B for 5 min, 5–15% B over 10 min, 15–45% B over 60 min, 45–70% B over 10 min, 70–100% over 5 min, and 100% B over 10 min). Separation was monitored at 214 nm.

CDR 01 – branco (fêmea/adulto) – Boa Vista

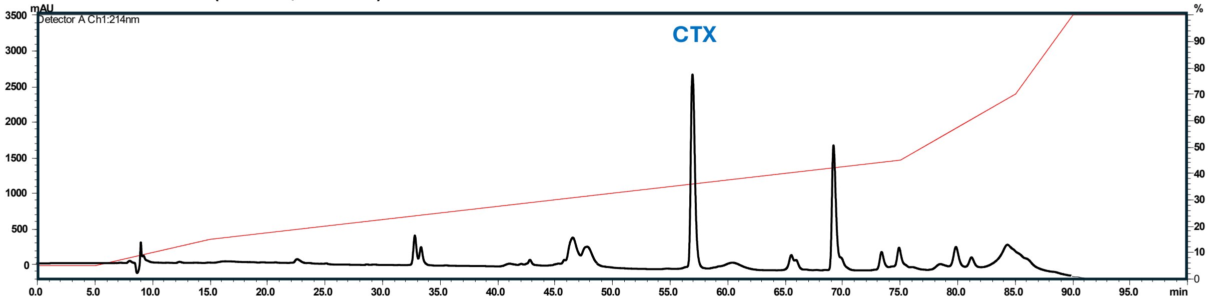

CDR 02 – amarelo (macho/adulto) - Bonfim

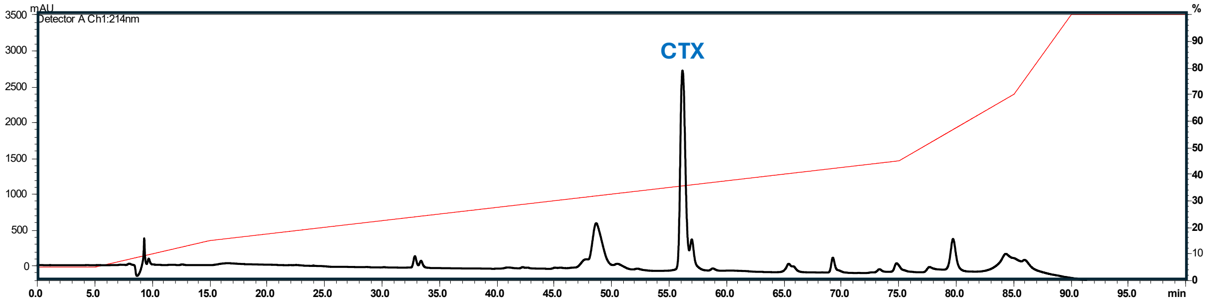

CDR 03 – branco (?/juvenil) - Cantá

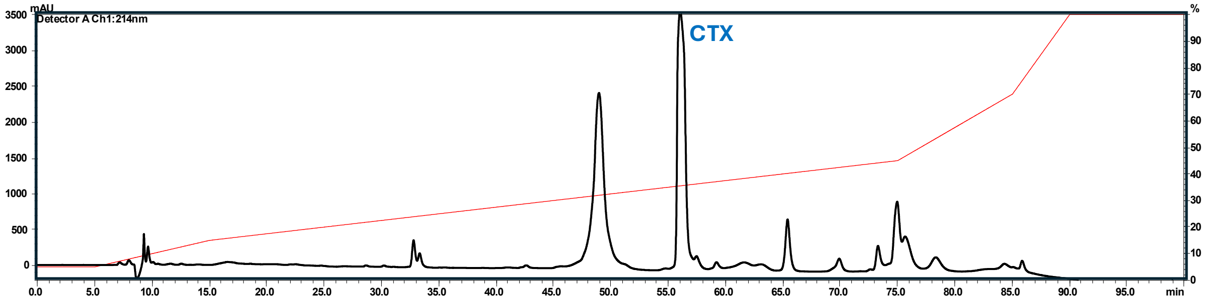

CDR 04 – amarelo (fêmea/adulto) - Bonfim

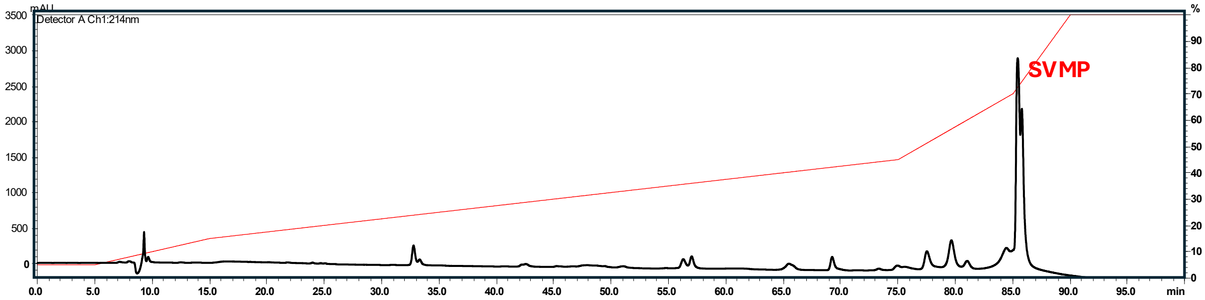

CDR 05 - MUSA -amarelo (fêmea/adulto) - Bonfim

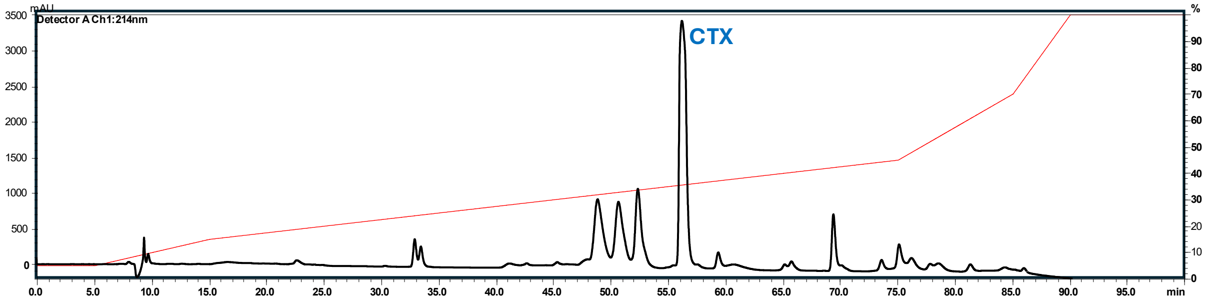

CDR 06 - MUSA -branco (macho/adulto) - Cantá

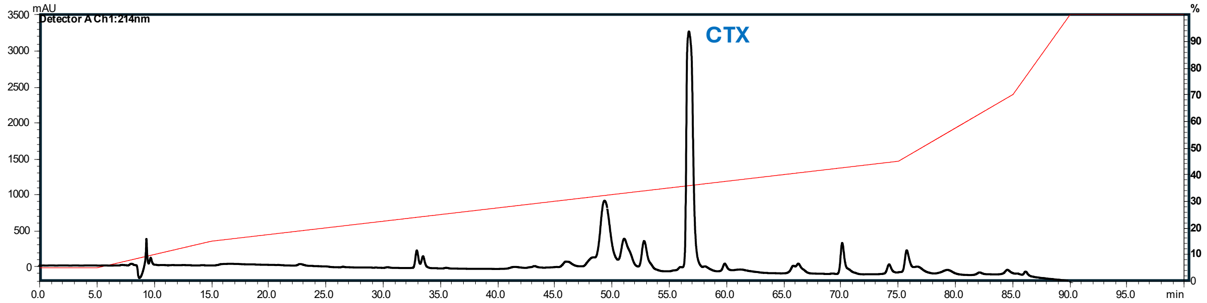

CDR 07 – branco (fêmea/adulto) - Normandia

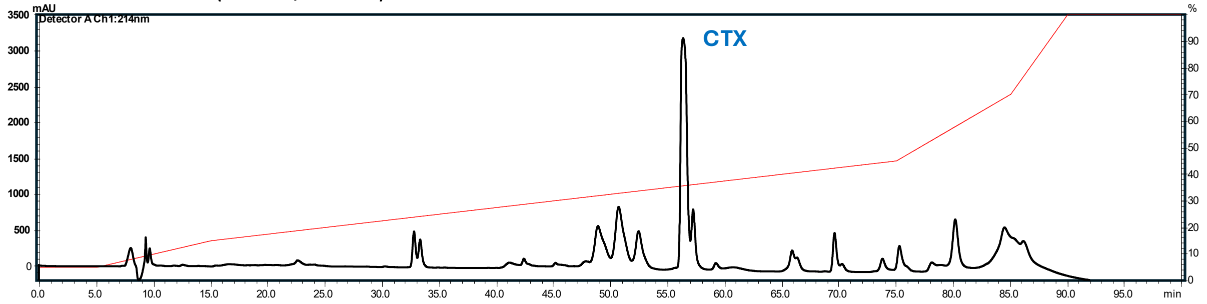

CDR 08 – amarelo (macho/adulto) - Bonfim

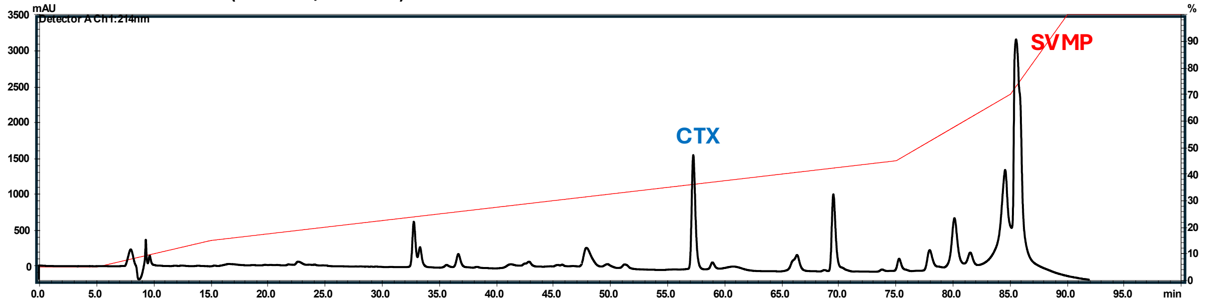

CDR 09 – amarelo (macho/adulto) – Bonfim

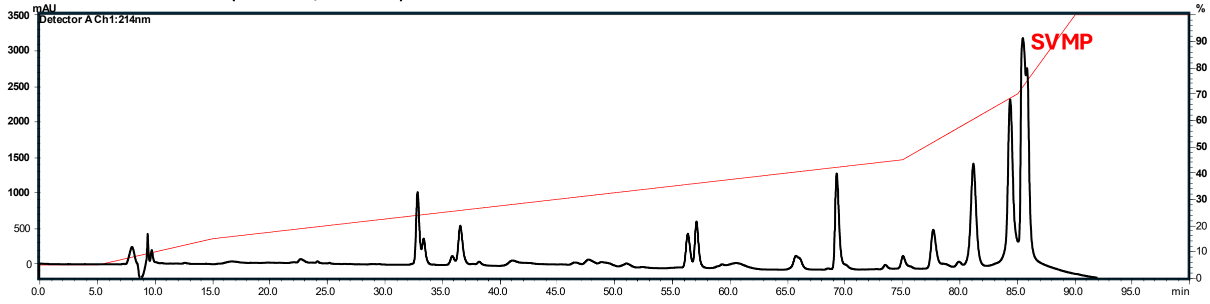

CDR 10 – branco (?/juvenil) - Bonfim

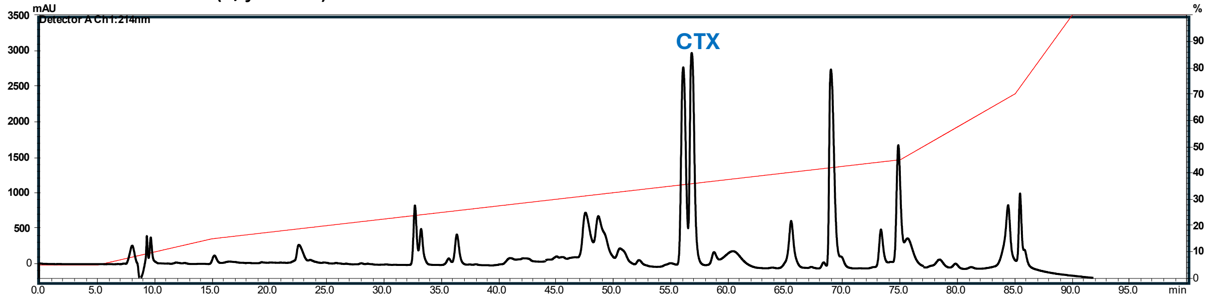

CDR 11 - amarelo (macho/adulto) – Bonfim

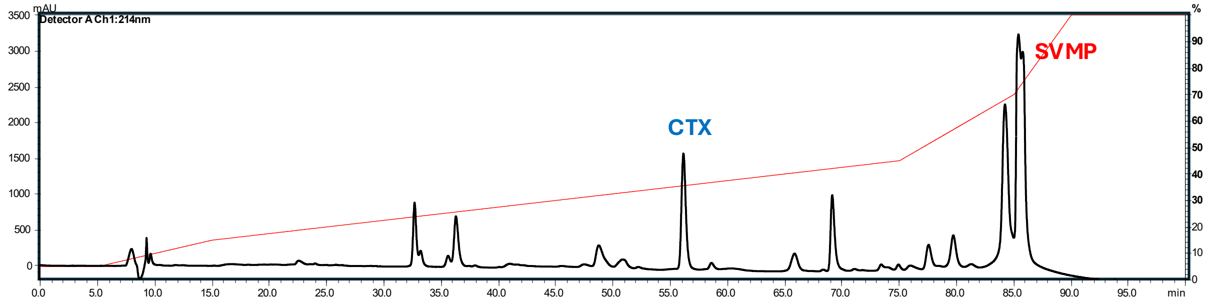

CDR 12 – amarelo (macho/juvenil) - Bonfim

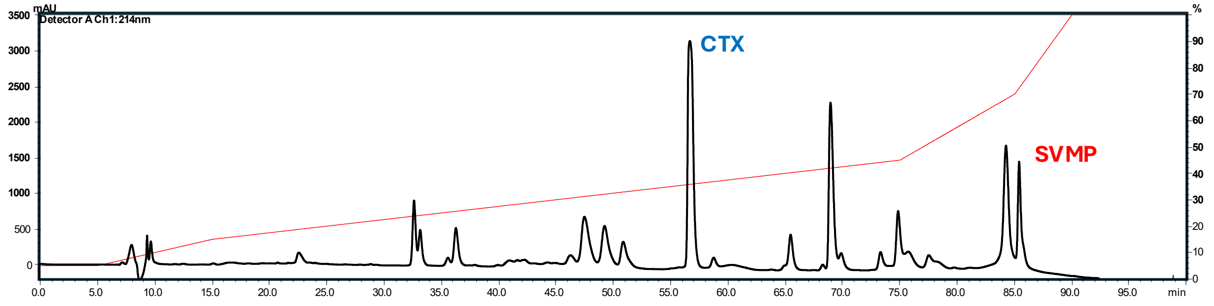

CDR 13 – amarelo (macho/adulto) – Bonfim

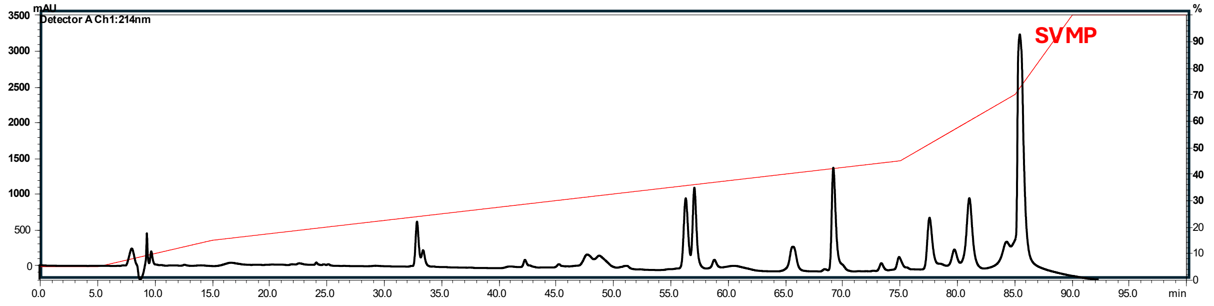

CDR SB0831 – amarelo – (macho/adulto) – Boa Vista

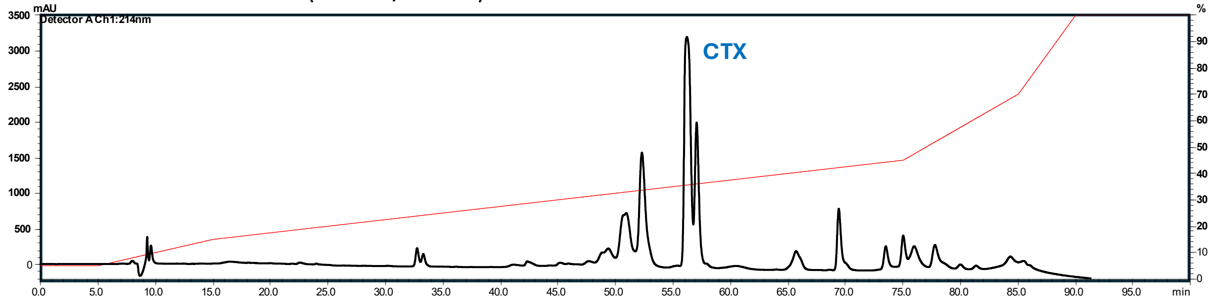

CDR SB0833 – amarelo – (macho/adulto) – Bonfim

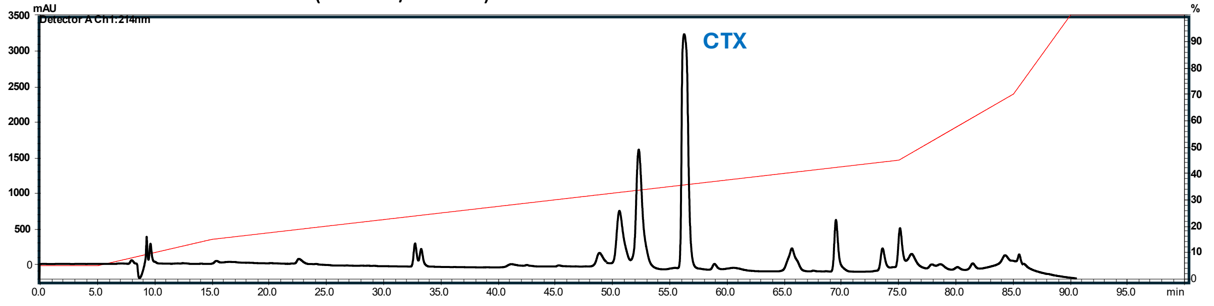

CDR SB0834 -branco (fêmea/adulto) – Boa Vista

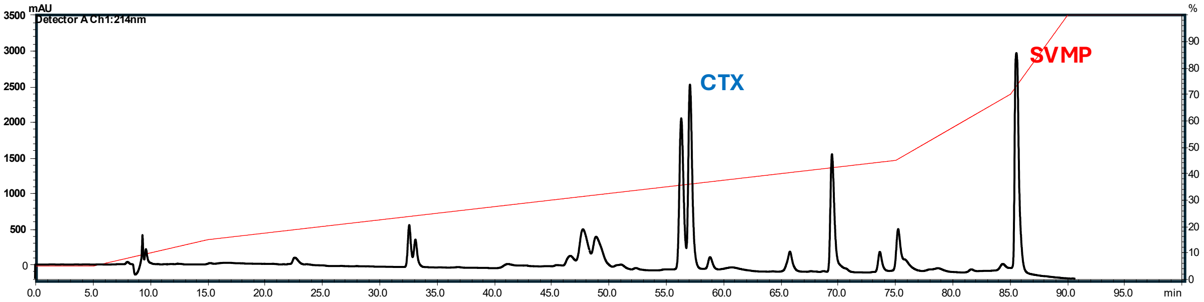

CDR SB1130 – amarelo – (?/adulto) – Boa Vista

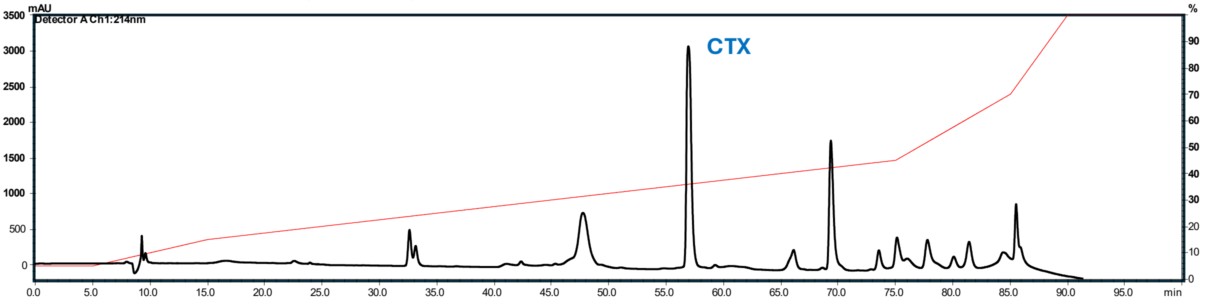

Crotalus durissus terrificus (pool Instituto Butantan)

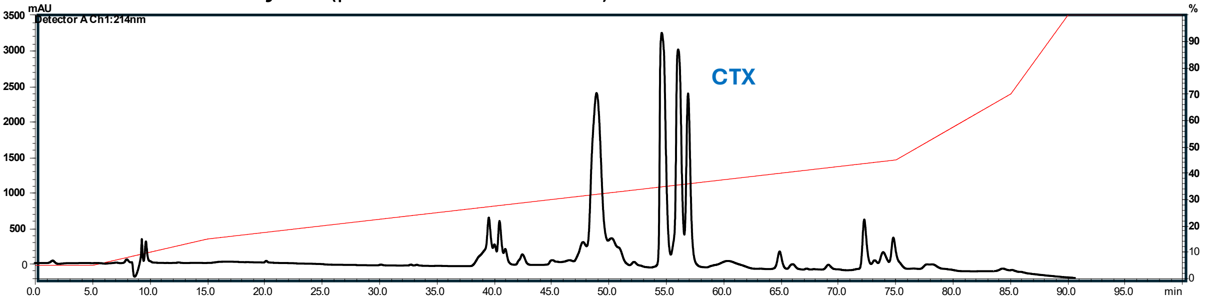

Supplement: S1 Fig — Individual venom samples (2 mg) were applied to a Phenomenex C-18 column. The mobile phases used were 0.1% TFA in water (solution A) or 0.1% TFA in acetonitrile (solution B). Proteins were eluted in a gradient at 2 mL/min (5% B for 5 min, 5–15% B over 10 min, 15–45% B over 60 min, 45–70% B over 10 min, 70–100% over 5 min, and 100% B over 10 min). Separation was monitored at 214 nm. (S1_Fig.PDF) [file pntd.0013296.s001.pdf]
