# Supplementary material for: Phenotypic dichotomy in Crotalus durissus ruruima venom and potential consequences for clinical management of snakebite envenomations
Source: PLoS Negl Trop Dis. 2025 Aug 1;19(8):e0013296. doi: 10.1371/journal.pntd.0013296 (PMC12327661; doi:10.1371/journal.pntd.0013296)
Supplement: S2 Fig — Annotation of the protein sequences predicted from the transcriptome of venom glands from SB831, SB833, SB834, and SB1130 specimens. (S2_Fig.PDF) [file pntd.0013296.s002.pdf]

Supplementary Figure 2: Master Seq of *Crotalus durissus ruruima* venom gland toxins

>CDR_PLA_01
MRALWIVAVLLVGVEGSLVEFETLMMKIAGRSGISYYSSYGCYCGAGGQGWPQDASDRCC
FEHDCCYAKLTGCDPTTDVYTYRQEDGEIVCGEDDPCGTQICECDKAAAICFRNSMDTYD
YKYLRFSPENCQGESQPC

>CDR_PLA_02
MRTLWIVAVLLLGVEGSLLDFEMMIIKVAKKSGLLWYSAYGCYCGWGGQGRPQDATDRCC
FVHDCCYGKVTDCDPKMVSYTYSVKNGEIICEDDDPCKKQTCECDGVAAVCFRDNIPSYD
KKYRQFPAENCREEPEPC*

>CDR_PLA_03
MRALWIVAVLLVGVEGHLLQFNKMIKFETRKNAIPFYAFYGCYCGWGGRGRPKDATDRCC
FVHDCCYGKLAKCNTKWDIYPYSLKSGYITCGKGTWCEEQICECDRVAAECLRRSLSTYK
YGYMFYPDSRCRGPSETC*

>CDR_PLA_04
MRTLWIVAVLLLGVKGSLVEFGRMIKEETGKNPFPSYTFYGCYCGLGGQGRPRDATDRCC
LMHDCCYENLTGCKTKTDPYPYSRKNRAIVCRGGTWCKKQICECDKAAAICLRDSLDTYN
KEYQFYGDFHCKEGPKKC

>CDR_SVMPII_01
MIQVLLVTICLAAFPYQGSSIILESGNVNDYEVIYPRKVTALPKGAVQPKYEDTMQYELK
VNGQPVVLHLEKNKGLFSKDYSETHYSPDGRKITTNPSVEDHCYYHGRIENDADSTASIS
ACNGLKGHFKLQGEMYIIEPLMFPDSEAHAVFKYENVEKEDEAPKMCGVTQNWESNEPIK
KASHLNLNPEHQRYVEIVIVVDHGMFTKYNGDSDKIRQRVHQMVNIMKESYSYMYIDISL
AGIEIWSNKDLINVQPAAPNTLKSFGEWRETDLLKRKSHDNAQLLTSIDFDGQTIGLAYI
GGICDSKRSTGVVMDHSKMNLRVALTMTHELSHNLGIHHDTGSCSCSGYSCIMSPVISDE
PSKYFSDCSYIQCWDFIMNQNPQCILKKPLRTDTVSTPVSGNELLEARIECDCGSIENPC
CYATTCKLRPGSQCAEGMCCDQCRFMKKGTVCRVSLVNKNDDTCTGQSADCPRNVLYG

>CDR_SVMPII_02
MIEVLLVTICLAAFPYQGSSVILESGNVNDYEVVYPRKVTALPKGAVQPKYEDAMLYELK
VNGEPVVLHLEKNKGLFSEDYSETHYSPDGREITTYPPVEDHCYYHGRIQNDADSTASIS
ACNGLKGHFKLQGEMYLIEPLKLPDSEAHAVFKYENVEKEDEAPKMCGVTQNWESYEPIK
KASHLNLNSEQQRYLNNFRYLELVIVVDYRMYTKYNGNLIIIRAWVYEIFNTINEIFQRM
NIHIALVGLEIWSHGDKIIVQSSADITLDLFGTWRATDLLSRKSHDNAQLLTSIDLDGPT
IGLAYIGGICDPKHSTGVVQDFSPINFLVAVTMAHEMGHNLGMTHDEYYCSCGGFACIMS
PVISPQPSKYFSDCSYIQYWIYINYHNPQCILNKPLRTDIVSPPVSGNELLEAGEECDCG
SPRNLCCDAATCKLRPGAQCAEGLCCDQCRFTRAGKICRRARGDNPDDRCTGQSADCPRN
RFHA

>CDR_SVMPII_04
MIQVLLVTICLAVFPYQGSSIILESGNVNDYEVVYPRKVTALPKGAVQPKYEDTMQYELK
VNGQPVVLHLEKNKGLFSKDYSETHYSPDGRKITTNPSVEDHCYYHGRIENDADSTASIS
ACNGLKGHFKLQGEMYIIEPLMFPDSEAHAVFKYENVEKEDEAPKMCGVTQNWESNEPIK
KASHLNLNPEHQRYVEIVIVVDHGMFTKYNGDSDKIRQRVHQMVNIMKESYSYMYIDISL
AGIEIWSNKDLINVQPAAPNTLKSFGEWRETDLLKRKSHDNAQLLTSIDFDGQTIGLAYI
GGICDSKRSTGVVMDHSKMNLRVALTMTHELSHNLGIHHDTGSCSCSGYSCIMSPVISDE
PSKYFSDCSYIQCWDFIMNQNPQCILKKPLRTDTVSTPVSGNELLEARIECDCGSIENPC
CYATTCKLRPGSQCAEGMCCDQCRFMKKGTVCRVSLVNKNDDTCTGQSADCPRNVLYG

>CDR_SVMPIII_01
MIQVLLVTICLAVFPYQGSSIILDSGNVNDYEVVYPRKVTALPKGAVQPKYEDTMQYEFK
VNGEPVVLHLEKNKGLFSEDYSETHYSPDGREITTYPPVEDHCYYHGRIQNDADSTASIS
ACNGLKGHFKLQGELYFIEPLKLPDSEAHAVYKYENVEKEDEAPKMCGVTQNWESYEPIK

KASQLNLTPEQRRYLSTKKYIELVIVADNVMVKKYTSNSTAIRTRIYACVNILNLIYRAF
NIHIALVGLEIWSNKDLIKVLSAAADTLKEFGDWRAADLLKRKKHDNAQLLTAIDLDRVI
GLAYVASMCDPKRSTGIVQDHSKLDVMVAVTMAHELAHNLGINHDGNQCNCNGNPCIMTE
KRSKPAYQFSNCSWDEHWRYLIKNRPSCILNKPLITDIVSPPVCGNYFVEMGEECDCGLP
ANCKNLCCNATTCKLKPGTQCEDGLCCDRCQLRRARNVCRPARSECDIAESCTGRSAECP
MDKFHRNGQPCLNNEGYCYNGTCPILDHQCISLFGSRATVAPDSCFDRNLQGQGNFYCRR
EKGKIFPCAPRDKKCGRLFCVQGPTGNKISCQSRAVPNDLNFGMVALGTKCGDGRVCNSN
RQCVDVNTAY

>CDR_SVMPIII_02
VNGEPVVLHLEKNKGLFSEDYSETHYSPDGREITTYPTIEDHCYYHGRIENDADSTASIS
ACNGLKGHFKLQGEIYLIEPLKLPDSAVHAAHAVYKYENVEKEDEAPKMCGVTQNWESYE
PIKKTSQSTLTPEQQRYLNAKKYVKLFLVADYIMYLKYGRNLTAVRTRMYDIVNVITPIY
HRMNIHVALVGLEIWSNTDKIIVQSSADVTLDLFAKWRATDLLSRKSHDNAQLLTGINFN
GPTAGLGYLGGICNTMYSAGIVQDHSKIHHLVAIAIAHEMGHNLGMDHDKDTCTCGTRPC
IMAGALSCEASFLFSDCSQKDHQEFLIKNMPQCILKKPLKTDVVSPAVCGNYFVEVGEEC
DCGSPRTCRDPCCDAATCKLRQGAQCAEGLCCDQCRFKGAGTECRAAKDECDMADVCTGR
STECTDRFQRNGQPCKNNNGYCYNGKCPIMADQCIALFGPGATVSQDACFQFNREGNHYG
YCRKEQNTKIACEPQDVKCGRLYCFPNSPENKNPCNIYYSPNDEDKGMVLPGTKCADGKA
CSNGQCVDVTTPY

>CDR_SVMPIII_03
MIQVLLVTICLAAFPYQGSSIILESGNVNDYEIVYPRKVTALPKGAVQPKYEDAMQYELK
VNGEPVVLHLEKNKQLFSKDYSETHYSPDGREITTYPLVEDHCYYHGRIENDADSTASIS
ACNGLKGHFKLQGEMYLIEPLKLSNSEAHAVYKYENVEKEDEAPKMCGVTQNWKSYEPIK
KASQLVVTAEHQKYNPFRFVELVLVVDKAMVTKNNDDLDKIKTRMYELANTVNDIYRYMY
IHVALVGLEIWSNEDKITVKPEAGYTLNAFGEWRKTDLLTRKKHDNAQLLTAIDLDRVIG
LAYVGSMCHPKRSTGIIQDYSPINLVVAVIMAHEMGHNLGIHHDSGYCSCGDYACIMRPE
ISPEPSTFFSNCSYFDCWDFIMNQNPECIVNEPMGTDIISPPVCGNELLEVGEECDCGTP
ENCQNECCDAATCKLKSGSQCGHGDCCEQCKFSKSGTECRASMSECDPAEHCTGQSSECP
ADVFHKNGQPCLDNYGYCYNGNCPIMYHQCYDLFGADVYEAEDSCFERNQKGNYYGYCRK
ENGNKIPCAPEDVKCGRLYCKDNSPGQNNPCKMFYSNEDEHKGMVLPGTKCADGKVCSNG
HCVDVATAY

>CDR_SVSP_01
MVLIRVLATLLILQLSYAQKSSELVVGGRPCNINEHRSLVVLFNSSGFLCAGTLINEEWV
LTAAHCKIKNFQIQLGVHSKKVRNEDEQTRDPKEKFFCLGSKTNNEWEKDIMLIRLNNPV
SNSAHIAPLSLPSSPPSVGSLCRIMGWGTISSTKEIYPNVPRCANINILDYAVCRAAYPW
WPVTTRILCAGILEGGKDSCQGDSGGPLICNGQFQGIVSWGADTCAQPREPGLYTKVFDY
TDWIKSIISGNTDATCPPPRENF

>CDR_SVSP_02
MVLIRVLATLLILQLSYAQKSSELVVGGRPCNINEHRSLVVLFNSSGFLCAGTLINQEWV
LTAAHCKIKNFQIQLGVHSKKVRNEDEQTRDPKEKFFCLGSKTNNEWEKDIMLIRLNNPV
SNSAHIAPLSLPSSPPSVGSLCRIMGWGTISSTKEIYPNVPRCANINILDYAVCRAAYPW
WPVTTRILCAGILEGGKDSCQGDSGGPLICNGQFQGIVSWGAHPCGRRLNPGFYTKVFDY
IDWIQSIIAGNTTVTCPQ

>CDR_SVSP_03
MGLIRVLANLLILQLSYAQKSSELVIGGDECNINEHRSLALVYITSGFLCGGTLINKEWV
LTAAHCDRGDILVLLGVHRLKDVQTGVSKDVQTRVAKEKFICPNRKKDDEKDKDIMLIRL
DSPVNISTHIAPLSLPSNPPSVGSVCRIMGWGAITSPNVTLPGVPHCADINIFDYEVCRA
AKPELPVTSRTLCAGILEGGKGSCDGDSGGPLICNGEIQGIVSWGGNICAQPREPGHYTK
VFDYTEWIQSIIAGNTDVTCPP

>CDR_SVSP_04
MVLIRVLANLLILQLSYAQKSSELVVGGDECNINEHRSLVAIFNSTEFFCSGTLINQEWV
VTAAHCDSTNFKMKLGVHSKKVPNEDEQTRNPKEKFFCPNKKKDDVLDKDIMLINLDSPV

SNSEHIMPLSLPSSPPSVGSVCHIMGWGSITPIEKTLPDVPYCANIKLLDDAVCKPPYPE
LPATSRTLCAGILEGGKDTCGGDSGGPLICNGQFQGIVFYGAHPCGQAPKPGVYTKVFDY
NDWIQSIIAGNTAATCPP

>CDR_SVSP_05
MVLIRVLANLLILQLSYAQKSSELVVGGDECNINEHRSLVVFFNSSGFLCAGTLINQEWV
LTAAHCDSKNFQMLFGVHSKKILNEDKQTRDPKEKFICPNRKKDDEKDKDIMLIRLDRPV
SNSEHIVPLSLPSSSPSVGSVCRIMGWGTISPTKKTYPDVPHCANINILDHLMCRAVYPE
LPATSRTLCAGILEGGKDTCRGDSGGPLICNEVIQGIVFGGGHPCGQPREPGVYTKVFDY
TDWIQSIIAGNTDATCPT

>CDR_SVSP_06
MVLIRVLANLLILQLSYAQKSSELVVGGHPCNINEHRSLVVLFNSSRFLCTGTLINEEWV
LTAAHCDSKNFQMQLGVHSKKVLNEDEQTRDPKEKFICPNKKKDDEKDKDIMLIRLDSPV
SNSEHIAPLSLPSSPPSVGSVCRIMGWGTISPTKETYPNVPHCANINILDHAVCRAAYPW
NPVTSTTLCAGSQQGGKDTCGADSGGPLICNGQFQGIVSWGGHPCGQAREPGVYVKVFDY
TEWIQSIIAGNTDATCPP

>CDR_SVSP_07
MVLIRVLANLLILQLSYAQKSSELIIGGEECNINEHRFLVALYTFRSKRFHCSGTLINQE
WVLTAAHCDRKNIRIKLGTHSTNVTNEDAQTRVPKEKFFCLSSKTYTRWDKDIMLIRLKR
PVNNSAHIATLSLPSSPPSVGSVCRIMGWGTISATKETYPDVPHCANINILDYEVCRAAH
GGLPATSRTLCAGILKGGKDSCKGDSGGPLICNGEIQGIVSWGAHPCGQSLKPGVYTKVF
DYIEWIQSIIAGNIDATCPP

>CDR_SVSP_08
MVLIRVLANLLILQLSYAQKSSELIFGGRPCNRNEHRFLALVYSDGNQCSGTLINEEWVL
TAAHCEGKKMKIHLGVHSKKVPNKDKQTRVAKEKFFCVSSKNYTKWNKDIMLIRLDRPVS
NSTHIAPLKLPSSSPSVGSVCRIMGWGTISPTEVILPDVPHCANINLLNYSECRAAYPEY
GLPATSRTLCAGILEGGKDTCVGDSGGPLICNGQFQGIASWGSTLCGYVREPGVYTKVFD
HLDWIQSIIAGNTDATCPL

>CDR_SVSP_09
MVLIRVLANLLILQLSYAQKSSELVIGGDECNINEHRFLVALYDPDRILCSGILLNEEWM
LTAAHCDRRDMLIKLGMHSKTVPNEDEQTRAPKEKFFCLSSKNYTLWDKDIMLIRLDSPV
SNSEHIAPLTLPSSPPSVGSVCRIMGWGRISPSKETYPDVPHCANINLLDYELCLAAYPE
FGLPATSKTLCAGILEGGKDSCKGDAGGPLICNGQFQGILSWGDDPCAQPRLPALYTKVF
DHLDWIQSIIAGNTDATCPFVNF

>CDR_SVSP_10
MVLIRVLANLLILQLSYAQKSSELVIGGDECNINEHRFLVALYDPDGFICGGTLLNEEWV
LTAAHCDKEKMEINLGVHSLNVVNEDEQTRVPKEKFFCLSSKNYTRWDKDIMLIRLDSPV
SNNERIAPFSLPSSPPSVGSVCHVMGWGRISPSKEIYPDVPHCANINILDYEVCRKAYPQ
FGLPVTSRTLCAGILEGGKDTCVSDSGGPLICNGQFQGIVSWGDDPCAQPHKPGVYTKVF
DHLDWIQSIVAGNTDATCPFVNF

>CDR_SVSP_11
MVLIRVLANLLILQLSYAQKSSELVIGGDEYNINEHRFLVALYHYRSHTFLCGGILINKE
WVLTAKHCDRKFMYIRLGMHNKNVKFDDDQRRSSKKKYFFRCHNNFTRWDKDIMLIRLNK
PVSYSEHIAPLSLPSSPPSVGSVCRVMGWGQTTSPQETLPDVPHCANINLLDYEVCRTAH
PQFGLPATSRILCAGVLEGGIDTCHRDSGGPLICNGQLQGIVSWGDGSCAEPDKPALYSK
VFDHLDWIQSIIAGNTTVNCPS

>CDR_SVSP_12
MVLIRVLANLLILQLSYAQKSSELVIGGDECNINEHNFLVALYEYWSQSFLCGGTLINGE
WVLTAAHCDRTHFLIYVGVHDRSVQFDKEQRRFPKEKYFFNCGNNFTKWDKDIMLIRLNK
PVRNNEHIAPLSLPSSPPSVGSVCRVMGWGQTTSPQNTLPDVPHCANINLLDYEVCRTAL
PQLRLPATSRILCAGVLEGGIDTCNRDSGGPLICNGEFQGIVFWGPDPCAQPDKPALYSK

VFDHLDWIQSIIAGNTIVNCPP

>CDR_SVSP_13
MVLIRVLANLLILQLSYAQKSSQLVIGGDECNINEHRFLAIVYTNSSQCAGTLINQEWVL
TAAHCDWENMDIYLGVHNESVQYDDEEGRVAAEKFFCLSSRNYIKWDKDIMLIRLNIPVR
NSTHIAPLSWPSSPPSVGSVCRVMGWGTITSPNETYPDVPHCANINLFDYEVCLAAYPEF
GLPATSRILCAGIQQGGKDTCGSDSGGPLICNGQFQGIVSWGDNPCAQPHKPALYTKVFD
YTEWIQSIIAGNTAVTCPP

>CDR_CTL_01
MGRFIFVSFGLLVLFLSLSGTGADFDCPSGWSAYDQYCYRVIKQLKTWEDAEWFCTKQAK
GAHLVSVESAGEADFVAQLVAENIKQNKYYVWIGLRIQNKGQQCSTKWSDGSSVNYENLL
KSYSKKCFGLKKETEFLQWYNTDCEEKNLFVCKFPPQC*

>CDR_CTL_02
MGRLVFVSFGLLVVFLSLSGTGADCPSGWSSYEGHCYKPFNELKTWDDAERFCTEQAKGG
HLVSIESAGEADFVAQLVADIARKGISYIWIGLRVQGEEKQCSTKWSDGSSISYENWIEE
ESKTCLGLEEDTNHKWVNIYCGQINPFVCKA*

>CDR_CTL_03
MGRLIFVSFGLLVVFLSLSGTAADCPSGWSSYEEHCYQIFHLFMTWKEAESFCKEQMKGG
HLVSIEDSEEADVMAEIVADNMRRTIPHIWIGLRRVQSKEKQCSTKWSDGSSVSYENWSV
AEFRTCLGLEQDTNYHKWANTFCGQRNPFVCKAKDPAV*

>CDR_CTL_04
MASFSLGLFLGLVLAPFTANAQADPCPSGWLAYNGHCYGYFEQEVNWQQAEAFCQSHNGH
LASIQTREEHQAVSNFLQKAQWWEHEDVWLGLFRPSHSQTWTWVDGTPVGYTAWEKHYRR
TWDSCAALEDSYGFLLWDDESCYDRNPFLCKTLAAAAPQGGQA*

>CDR_CTL_05
MGRFIYVSLGLLVVAFSLRGSGADCPSGWSSYDKNCYKVFDERKNWDEAQRFCMEHGKEG
HLVSPGSNEEGNFVAKLTSNEFKERHPAYVWIALSAQDQEKLLRTTCHGKHGPVPCGPTL
ELCIVNGEVIPCILTKPPPLRSRQCLVLSKRTGYLKWNNHQCGFKFPFVCKFLAAPDDSE

>CDR_CTL_06
MGRFIYVSLGLLVLAFSLRGSGADCPSGWSSYDKNCYKVFDERKNWDEAERFCTEQGKEG
HLVSLGRIEEGNFVAKLTFQELKKPHPTYLWIGLTAQGQEKQMAKCKKYWREQRCRPLLS
KTGLVSSKQTENLKWNNHNRGFTLPFVCKFLAEPEDLE

>CDR_CTL_07
MGRFIYVSLGLLILAFSLRGSGADCPSGWSSYDKNCYKVFDERKNWDEAERFCTEQGKEG
HLVSLGSVEEGNFVAKLTVQELKKSHPTNVWIELTAQGLTQHSRGSHNYCRNGYKWPLCC
PKNIRKCLVLSKHTENLKWKNHNRGFTVPFVCKFLAEPDDLE

>CDR_CTL_08
MGRFIFVSFGLLVVFLSLSGTGAGFCCPLGWSSYDQHCYKVFSELKTWDDAESFCYTQHR
GSRLASIHSSEEEAFVGKLASQTLKFTSMWIGLKDLWKECNWQWSDDTKLDYKAWTRRPY
CTVMVVKTDRIFWFNRGCEKTVSFVCKFQA

>CDR_CTL_09
MGRFIFVSFGLLVVFLSLSGSEAGFCCPSHWSSYDRYCYKVFKQEMTWADAEKFCTQQHT
GSHLVSFHSTEEVDFVVKMTHQSLKSTFFWIGANNIWNKCNWQWSDGTKPEYEEWHEEFE
CLISRTFDNQWLSAPCSDTYSFVCKFEA*

>CDR_CTL_10
MATGTGAGFCCPLGWSSYEGHCYKVFKQDMTWEDAEKFCTQQHEGSHLVSLQSSEEVDFV
ISMTAPMLKLGLVWIGLSNIWNECTLEWTNGNKVDYKAWSAEPECIVSKSTDKHWFSRPC
SKTHKVVCKFQA*

>CDR_CTL_11
MGRFIFVSFGLLVVFLSLSGTAAVCPSGWSSYEGHCYKPFNEAKNWDDAENFCTQQHTGG
HLVSFHSTEETDFVGKLAFQTFGQSIFWIGLSNVWNKCSWQWSNGAMLKYEDWAEESYCV
YFKSTNNKWRSRACRMMAHFVCEFQA

>CDR_CTL_12
MGRFIFVSFGLLVVFLSLSGTAAVCPSGWSSYEGHCYKPFNEAKNWDDAENFCTQQHTGG
HLVSFHSTEETDFVGKLSFQTFGQSIFWIGLSNVWNKFLLSVTGSISSPALLGFQLLEEA
PLCPKEGGRFPGVELSSWGLCEEKAMQLAMEQWCHAEIRRLG

>CDR_CTL_13
MGRFICVSFGLLVVFLSLSGTGADFDCPSGWSAYDRYCYKPFNEPQNWDDAERFCSEQAK
GAHLVSIESDGEADFVAQLAQKIDKPDIYVWIGLRVQGKEQQCSTKWSDGSSIIYVNWNK
GESQMCQGLSRWTNFLKWDYTDCQAKKPFVCKFPPEC

>CDR_CTL_14
MGRFIFVSFGLLVLFLSLSGTGAGLHCPSDWYYYDQHCYRIFNEEMNWEDAEWFCTKQAK
GAHLVSIKSAKEADFVAWMVTQNIEESFSHVWIGLRVQNKEKQCSTKWSDGSSVSYDNLL
DLYITKCSLLKKETGFRKWFVVSCIGKIPFVCKFPPQC

>CDR_CRISP_01
MIAFIVLPILAAVLQQSSGSVDFDSESPRKPEIQNKIVDLHNSLRRSVNPTASNMLKMEW
YPEAAANAERWAYRCIESHSPHDSRVLGEIKCGENIYMSSVPIKWTEIIHGWHGENKNFK
YGIGAVPPNAVTGHFSQIVWYKSYRVGCAAAYCPSSKYSYFYVCQYCPAGNIIGKTATPY
KSGPPCGDCPSACDNGLCTNPCTKEDKYTNCKSLVQQAGCQDKQMQSDCSAICFCQNKII

>CDR_Kunitz_01
MRREKSLALLITLAAALAAAETLPDLCHSSKMVGPCRASFHRWWYNATSQTCQEFIFGGC
RGNANNFFSEQDCFQTCARGGGVEATVVPGRLATEVATSRGGRHPEAYENRPGFREFCAA
PRVVGPCRASFHRWYFDLESRTCKMFVYGGCRGNKNNYLFEEHCWSQCTGDGEITEEPGD
TDAHPRILSEPFSFSTRAVVLAVLLAILVAILLGSMVVFFVKICRKNPELSVGTVWSTLD
DKEYLMSNAYSL*

>CDR_BPP_01
MFVSRLAASGLLLLLALLAVSLDGKPLQQWSQRWPHPQIPPLVVQNWKSPTQLQARESPAG
GTTALREELSLGPEAALDTPPAGPDGGPRGSKAAAAAPQRLSKSKGASATSAASRDLRTD
GKQARQNWGRLVSPDHHSAAGGGGGGGGGARRLKGLAKKRAGNGCFGLKLDRIGSMSGLG
C*

>CDR_NUC_01
MQTPKRRRGAQGCPRSSPSQPLLLLVRAVWFCAALSVAAGSFELTILHTNDVHARVEQTS
RDSGKCTGQDCYGGVARRATKIRELRAKHRHVLLLDAGDQYQGTVWFNFFKGREVVKFMN
SLRYDAMALGNHEFDNGLAGLLDPLLKHANFPILSANIRPKGSIASNISGYILPYKIINV
GSEKVGIIGYTTKETPVLSNPGPYLEFRDEVEELQNHANKLTTLGVNKIIALGHSGFSED
QRIARKVKGVDVVVGGHTNTFLYTGSPPSTEVAAGNYPFMVQSDDGRQVPVVQAYAFGKY
LGYLNVIFDDKGNVIKSSGNPILLNKDISEDQDIKAEVNKMKIQLHNYSSQEIGKTIVYL
NGTTQACRFHECNLGNLICDAVIYNNVRHPDDNEWNHVSMCIVNGGGIRSPIDERANNGT
ITLEELTAVLPFGGTFDLLQIKGSALKQAFEHSVHRHGEGMGELLQVSGIKVVYDLSRKP
GSRVLSLNVLCTECRVPTYVPLEKEKTYKLLLPSFLAAGGDGYHMLKGDSSNHSSGNLDI
SIVGDYIKRMGKVFPAVEGRMIFSAGTLFQAQLFLTWGLCISLLYFIL

>CDR_PLB_01
MIRFGNPSSSDKRRQRCRSWYWGGLLLLWAVAETRADIHYATVYWLEAEKSFQIKDVLDK
NGDAYGYYNDTIQSTGWGILEIKAGYGNQPVSNEILMYAAGFLEGYLTASHMSDHFANLF
PLMIKNVIIEQKVKDFIQKQDEWTRQQIKNNKDDPFWRNAGYVIAQLDGLYMGNVEWAKR
QKRTPLTDFEISFLNAIGDLLDLIPALHSELRKSDFRSMPDVSRIYQWDMGHCSALIKVL
PGYENIYFAHSSWFTYAATLRIYKHLDFKITDPQAKTGRASFSSYPGFLVSLDDFYILDS

GLIILQTTNSVFNLSLLKKVVPESLFAWERVRIANMMADSGKTWAETFEKQNSGTYNNQY
MILDTKKIKLQRSLEDGTLYIIEQVPKLVKYSDQTKVLRNGYWPSYNIPFDKEIYNMSGY
GEYVQRHGLEFSYEMAPRAKILRRDQGKVTDMESMKFIMRYNNYKEDPYAKRNPCNTICC
RQDLDRRTPVPAGCYDSKVADISMAAKFTAYAINGPPVEKGLPVFSWVHFNKTKHQGLPE
SYNFDFVTMKPVL*

>CDR_LAAO_01
MNVFFMFSLLFLAALGSCAHDRNPLEECFRETDYEEFLEIARNGLTVTSNPKHVVIVGAG
MAGLSAAYVLAGAGHQVTVLEASERVGGRVRTYRKKDWYANLGPMRLPTKHRIVREYIRK
FGLQLNEFFQENENAWYFIKNIRKRVREVKNNPGILEYPVKPSEEGKSAAQLYVESLRKV
VKELKRTNCKYILDKYDTYSTKEYLLKEGNLSPGAVDMIGDLLNEDSGYYVSFIESLKHD
DIFGYEKRFDEIVGGMDQLPTSMYEAIKEKVQVHFNARVIEIQQNDRETKVTYQTSANEM
SSVTADYVIVCTTSRAARRIKFEPPLPPKKAHALRSVHYRSGTKIFLTCKRKFWEDDGIR
GGKSTTDLPSRFIYYPNHNFTSGVGVIIAYGIGDDANFFQALDFKDCADIVINDLSLIHQ
LPKEDIQTFCRPSMIQRWSLDKYAMGGITTFTPYQFQHFSEALTAPFKRIYFAGEYTAQF
HGWIDSTIKSGLTAARDVNRASENPSGIHLSNDNEF*

>CDR_HYAL_01
MYHLWIKCLAAWIFLKRFNGVHVMQAKAPMYPNEPFLVFWNAPTTQCRLRYKVDLDLNTF
HIVTNANDSLSGSAVTIFYPTHLGFYPHIDGRGHFFNGIIPQNESLAKHLNKSKSDINRM
IPLRTFHGLGVIDWENWRPQWDRNWGSKNVYRNRSIQFARDLHPELSEDEIKRLAKQEYE
KAAKSFMRDTLLLAEEMRPDGYWGYYLYPDCQNYNYKTKPDQYTGECPDIEMSRNNQLRW
LWRDSTALFPNIYLETVLRSSDNALKFVHHRLKESMRIASMARKDYALPVFPYARPFYAY
TFEPLTEEDLVNTVGETAAMGAAGIVFWGSMQYASTVDSCRKVKDYMDGPLGRYIVNVTT
AAKICSHFLCKKHGRCVRKHSDSNAFLHLFPDSFRILVHGNATEKKVIVKGKLELENLIF
LINNFMCQCYQGWKGLYCEKHSIKDIRKI*

>CDR_Warprin_01
MTPRRGSCPLLLFSLVGLLVTCAQEPDTARQNTTAAAEKAGTCPQAELETPNGNCTEECQ
SDASCEGNQKCCQTGCGTSCQIPDGKPGSCPNVDMPIPPLGVCRDMCKTDSSCADKMKCC
KNGCGFMTCSNPVP*

>CDR_PDE_01
MIQQKVLFISLVAVTLGLGLGLGLKEPVQPQAQSWSCSKLRCGEKRIANVLCSCSEDCLE
KKDCCTDYKTICKGETSWLKDKCASSGATQCPAGFEQSPLILFSMDGFRAGYLENWDSLM
PNINKLKTCGTHAKYMRAVYPTKTFVNHYTIATGLYPESHGIIDNNIYDVNLNLNFSLSS
STARNPAWWGGQPIWHTATYQGLKAATYFWPGSEVKINGSYPTIFKNYNKSIPFEARVTE
VLKWLDLPKAKRPDFFTLYIEEPDTTGHKYGPVSGEIIKALQMADRTLGMLMEGLKQRNL
HNCVNLILLADHGMEEISCDRLEYMANYFNNVDFFMYEGPAPRIRSKNVPKDFYTFDSEG
IVKNLTCRKPKQYFKAYLSKDLPKRLHYANNIRIDKVNLMVDQQWMAVRDKKFTRCKGGT
HGYDNEFKSMQAIFLAHGPGFNEKNEVTSFENIEVYNLMCDLLKLKPAPNNGTHGSLNHL
LKNPFYTPSPAKEQSSPLSCPFGPVPSPDVSGCKCSSITELEKVNQRLNLNNQAKTESEA
HNLPYGRPQVLQNHSKYCLLHQAKYISAYSQDILMPLWSSYTIYRSTSTSVPPSASDCLR
LDVRIPAAQSQTCSNYQPDLTITPGFLYPPNFNSSNFEQYDALITSNIVPMFKGFTRLWN
YFHTTLIPKYARERNGLNVISGPIFDYNYDGHFDSYDTIKQHVSNTKIPIPTHYFVVLTS
CENQINTPLNCLGPLKVLSFILPHRPDNSESCADTSPENLWVEERIQIHTARVRDVELLT
GLNFYSGLKQPLPETLQLKTFLPIFVNPVN*

>CDR_NGF_01
MSMLCYTLIIAFLIGIWAAPKSEDNVPLGSPATSDLSDTSCAKTHEALKTSRNIDQHYPA
PKKAEDQEFGSAANIIVDPKLFQKRRFQSPRVLFSTQPPPLSRDEQSVDNANSLNRNIRA
KREDHPVHKRGEYSVCDSVNVWVANKTTATDIRGNLVTVMVDVNINNNVYKQYFFETKCR
NPNPVPTGCRGIDARHWNSYCTTTNTFVKALTMEGNQASWRFIRIDSACVCVISRKNENF
G*

>CDR_VEGF_01
MHLLGLFSLGYYLAAAAVVLLLAREPAIAAAYESGQGYYEEEPDLGEAKNYENKELEEQL
RSVSSVDELMTVLYPEYWKMFKCQLRKGGWPHREQSNFDGRTGDSNPIKFAAAHYNTEIL

KSIDNEWRKTQCMPREVCVDVGKESGATTNIFFKPPCVSVYRCGGCCNSEGLQCMNISAS
YVSKTLFEITVPLSHGPKPVTISFANHTSCRCMSKLDVYRQVHSIIRRSLPVIQAQCQIT
NKTCPKSLIWNNHLCKCLAQHDFSFSSYPPDTDTAEGYHDICGSNKELDEETCQCVCKGG
VTPSSCGIHKELDRTSCQCTCKNKLLPSSCGLNKEFDEEKCQCVCKRTCPKHQPLNPTKC
ICECVESPNKCFLKGERFHHQTCSCYRRPCTVRKKRCDSGFYYSEEVCRCVPTYWKLPLI
N*

>CDR_VEGF_02
MAAYLLAVAILFCIQGWPSGTVQGQVMPFMEVYERSVCQTREMLVSILDEYPSEVAHLFR
PSCVTVLRCGGCCTEESLTCTATGKRSVGREIMRVDPRQGTSKIEVMQFTEHTECECRPG
STVNNGKRKKNPKEGEPRAKFPLV*
