# Supplementary figures and images for: Phenotypic dichotomy in Crotalus durissus ruruima venom and potential consequences for clinical management of snakebite envenomations
Source: PLoS Negl Trop Dis. 2025 Aug 1;19(8):e0013296. doi: 10.1371/journal.pntd.0013296 (PMC12327661; doi:10.1371/journal.pntd.0013296)

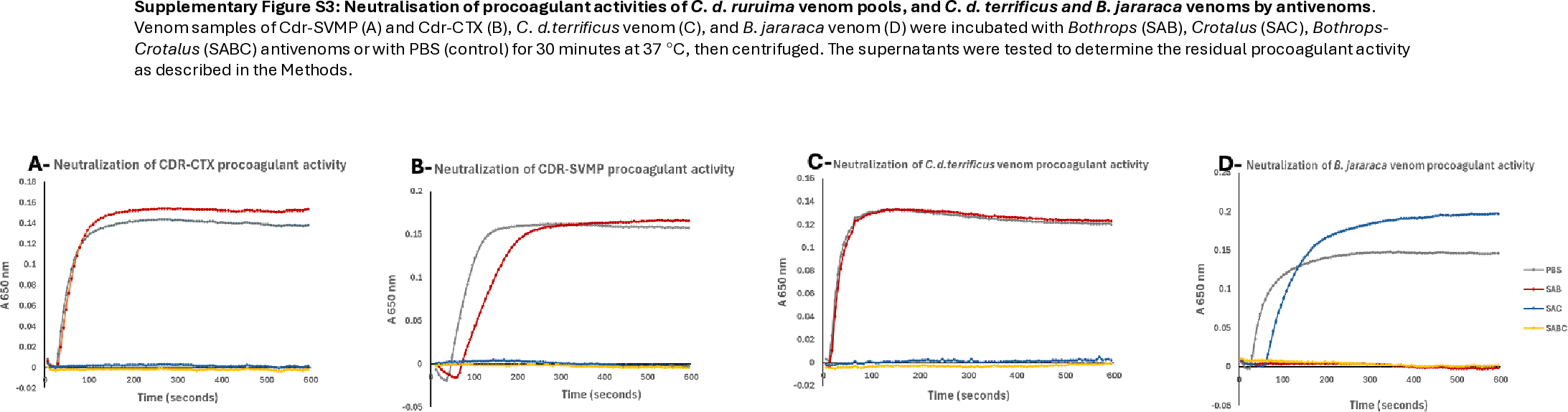

Supplement: S3 Fig — Venom samples of Cdr-SVMP (A) and Cdr-C TX (B), C. d. terrificus venom (C), and B. jararaca venom (D) were incubated with Bothrops (SAB), Crotalus (SAC), Bothrops-Crotalus (SABC) antivenoms or with PBS (control) for 30 minutes at 37 °C, then centrifuged. The supernatants were tested to determine the residual procoagulant activity as described in the Methods. (S3_Fig.TIF) [file pntd.0013296.s003.tif]
